# Supplementary material for: Understanding self-assembly at molecular level enables controlled design of DNA G-wires of different properties
Source: Nat Commun. 2022 Feb 25;13:1062. doi: 10.1038/s41467-022-28726-6 (PMC8881451; doi:10.1038/s41467-022-28726-6)
Supplement: Supplementary file 1 — Supplementary information [file 41467_2022_28726_MOESM1_ESM.pdf]

SUPPLEMENTARY INFORMATION

Understanding self-assembly at molecular level enables controlled design of DNA G-wires of different properties

Daša Pavc[1,2], Nerea Sebastian[3], Lea Spindler[4,3], Irena Drevenšek-Olenik[5,3], Gorazd Koderman Podboršek[6,7], Janez Plavec[1,2,8], Primož Šket[1,*]

[1]Slovenian NMR Centre, National Institute of Chemistry, SI-1000 Ljubljana, Slovenia

[2]University of Ljubljana, Faculty of Chemistry and Chemical Technology, SI-1000 Ljubljana, Slovenia

[3]Department of Complex Matter, Jožef Stefan Institute, SI-1000 Ljubljana, Slovenia

[4]University of Maribor, Faculty of Mechanical Engineering, SI-2000 Maribor, Slovenia

[5]University of Ljubljana, Faculty of Mathematics and Physics, SI-1000 Ljubljana, Slovenia

[6]Department of Materials Chemistry, National Institute of Chemistry, SI-1000 Ljubljana, Slovenia

[7]Jožef Stefan International Postgraduate School, SI-1000 Ljubljana, Slovenia

[8]EN-FIST, Center of Excellence, SI-1000 Ljubljana, Slovenia

Correspondence and requests for materials should be addressed to P.Š. (email: primoz.sket@ki.si).

Table of contents

1. SUPPLEMENTARY FIGURES 2

1.1 Schematic presentation of possible structural types of G-wires 2

1.2 d($G_2AG_4AG_2$) G-wires surface self-assembly 3

1.3 Scanning for optimal solution conditions 6

1.4 Structural characterization of Q1k 8

1.5 Structural characterization of Q2k 12

1.6 Identification and characterization of Q2t 14

1.7 Characterization of Q4t 18

1.8 Phase diagram of d($G_2AG_4AG_2$) self-assembly 19

1.9 Characterization of G-wires with thymines and cytosines in loops 20

1.10 G-wires thermal stability studies 27

1.11 G-wires with c3 linkers and abasic residues in loops 31

1.12 Structural ensembles of model Q4t G-quadruplexes 32

1. Supplementary figures

1.1 Schematic presentation of possible structural types of G-wires

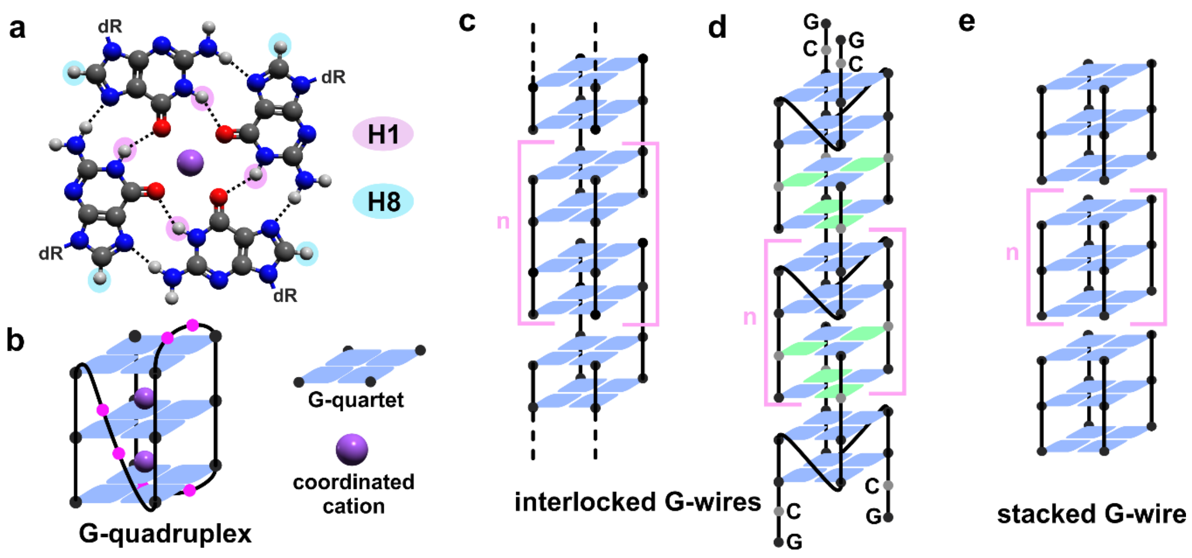

Supplementary Figure 1. Schematic presentation of G-quartet, G-quadruplex and different structural types of G-wires. a G-quartet is formed by four guanine residues in planar arrangement interconnected via Hoogsteen hydrogen bonds. H1 and H8 protons of guanine residues within G-quartet are shadowed with pink and blue, respectively. Cations, which are coordinated between/within G-quartets, assist their formation and stacking into **(b)** G-quadruplex structures. Pink circles present loop residues. G-wires can be formed by interlocking of G-quadruplexes via **(c)** slipped G-rich strands and **(d)** sticky ends (e.g., GC-ends) as well as via **(e)** π-π stacking of G-quadruplexes. Blue and green rectangles represent guanine and cytosine residues, respectively. **c-e** Pink brackets show repeating G-quadruplex building block in each G-wire type.

1.2 d($G_2AG_4AG_2$) G-wires surface self-assembly

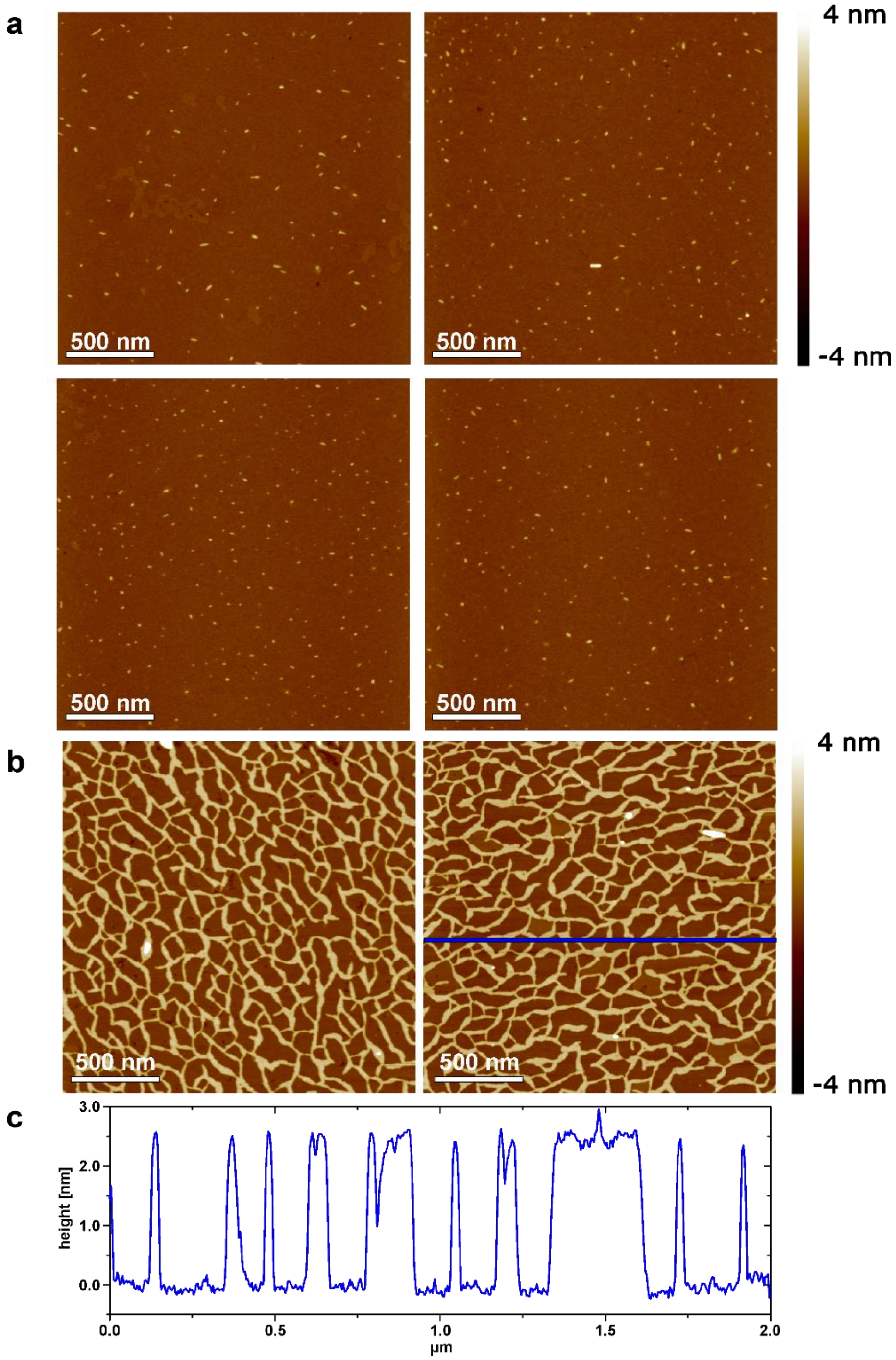

Supplementary Figure 2. AFM images of d($G_2AG_4AG_2$) G-wires. a Characteristic AFM height images at low deposition concentrations (0.2 µM) enable visualization of individual G-wires. **b** Characteristic AFM height images observed for higher sample concentration deposition (2 µM) showing a network pattern of 2.4 nm height. **c** Height profile along blue line in **(b)**. Sample was assembled via method 1 (Methods).

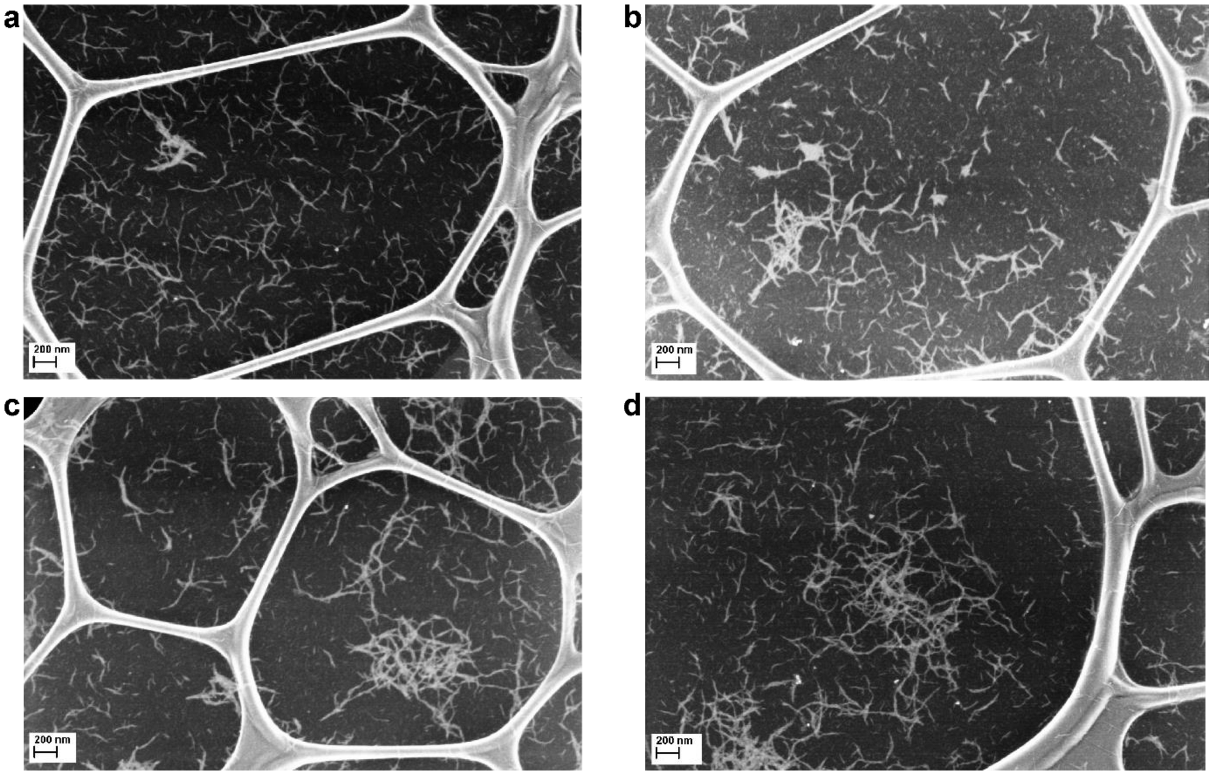

Supplementary Figure 3. SEM images of d($G_2AG_4AG_2$) G-wires. Spots where we observe **(a,b)** more individual and **(c,d)** bigger deposits of G-wires. Sample deposition concentration was 1.0 mM. Sample was assembled via method 1 (Methods).

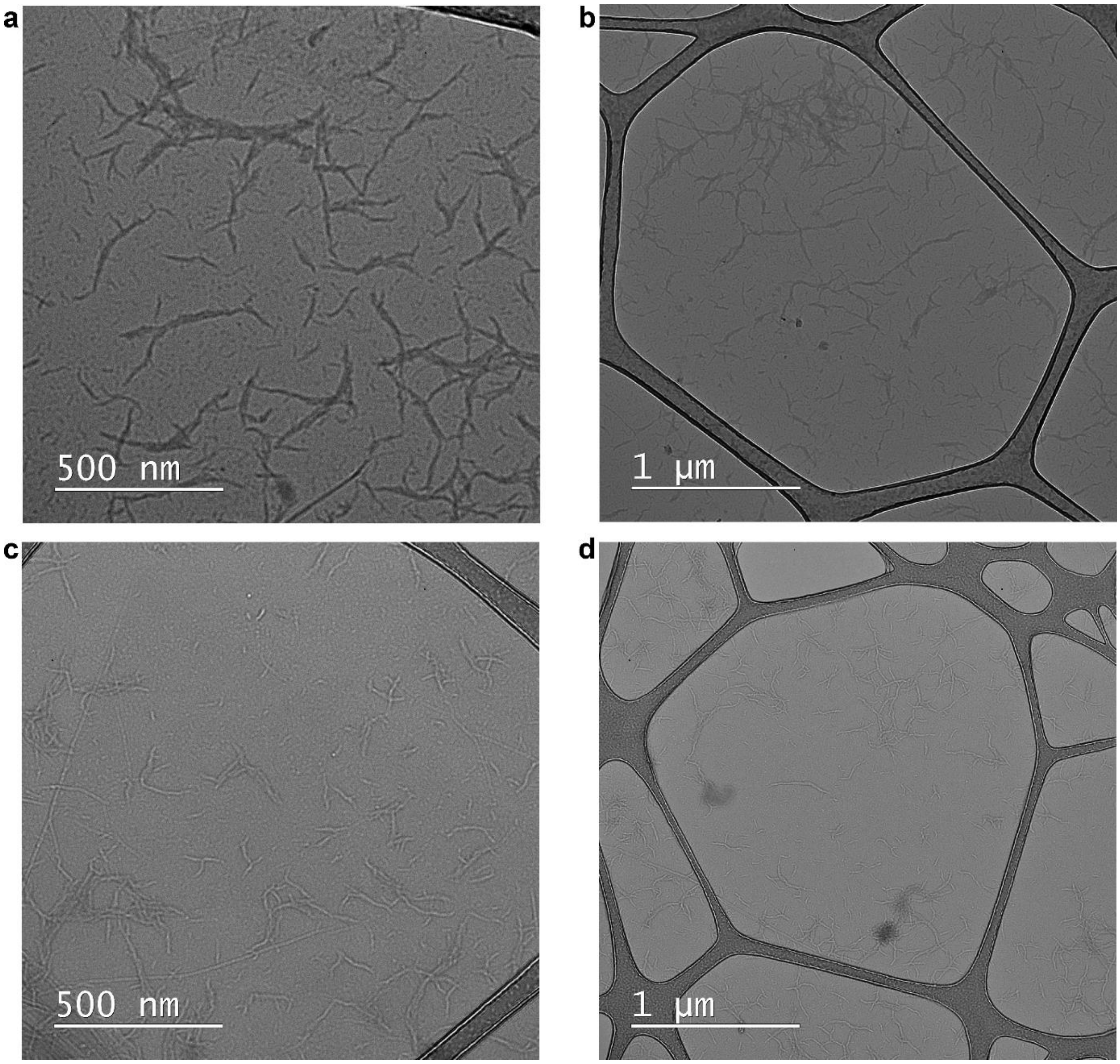

Supplementary Figure 4. Conventional TEM images of unstained d($G_2AG_4AG_2$) G-wires. a,b TEM images were taken at substantial under-focus and **(c,d)** over-focus. Spots where we observe **(a,c)** more individual and **(b,d)** bigger deposits of G-wires. Sample deposition concentration was 1.0 mM. Sample was assembled via method 1 (Methods).

1.3 Scanning for optimal solution conditions

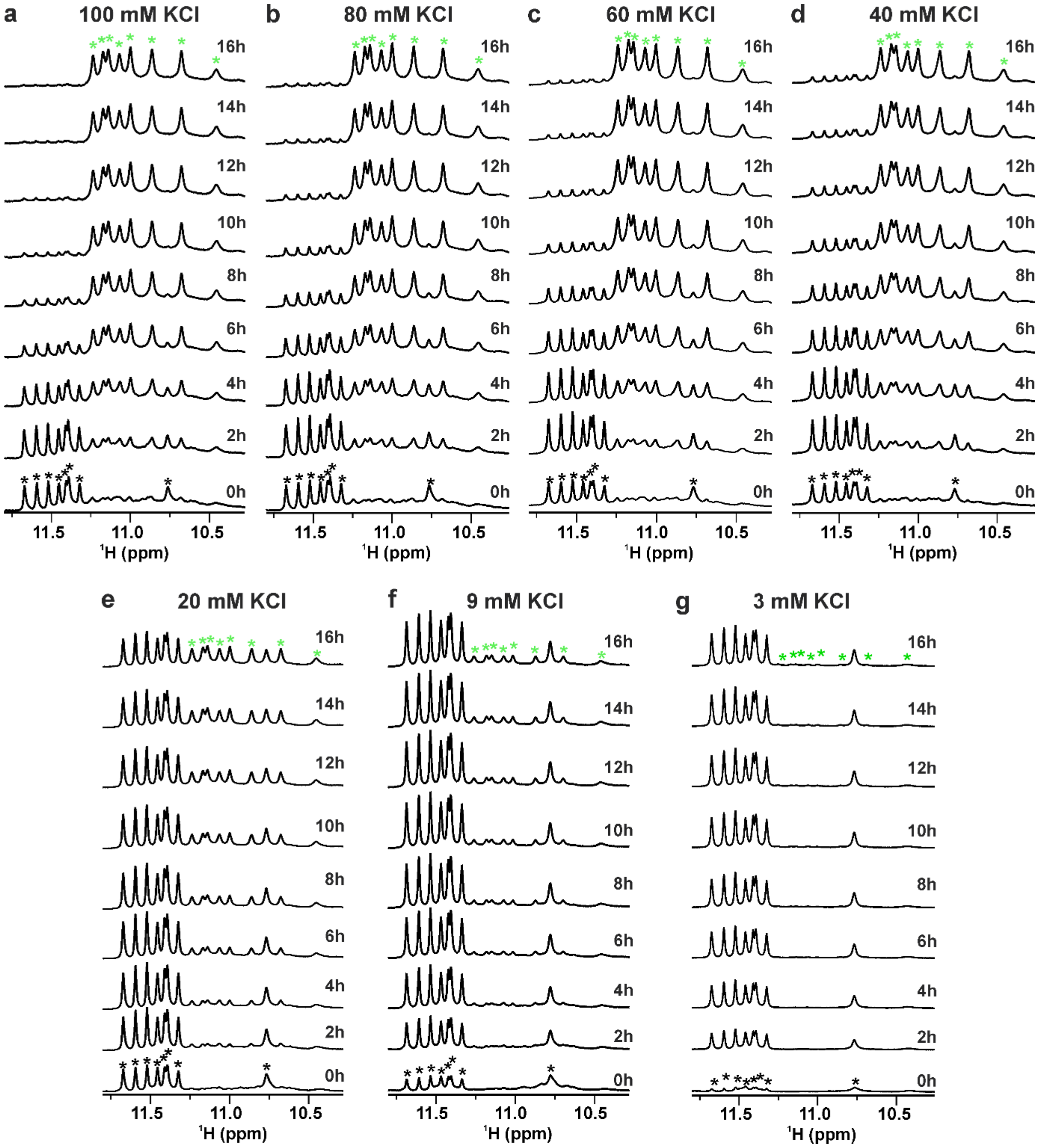

Supplementary Figure 5. Equilibrium between Q1k and Q2k depends on KCl concentration. H1 regions of a set of 1D ^{1}H NMR spectra acquired in **(a)** 100, **(b)** 80, **(c)** 60, **(d)** 40, **(e)** 20, **(f)** 9 and **(g)** 3 mM KCl concentrations as indicated above individual set of spectra. Black and green asterisks denote H1 signals corresponding to Q1k and Q2k, respectively. Spectra were acquired every 15 min over the course of 17h starting immediately after the addition of KCl into oligonucleotide solution at 0°C (PAD experiments, Methods). For clarity, only selected spectra are shown. Spectra were acquired at 800 MHz, 0°C, 1.0 mM oligonucleotide concentration in 90% H_2O/10% D_2O. Samples were assembled via method 2 (Methods).

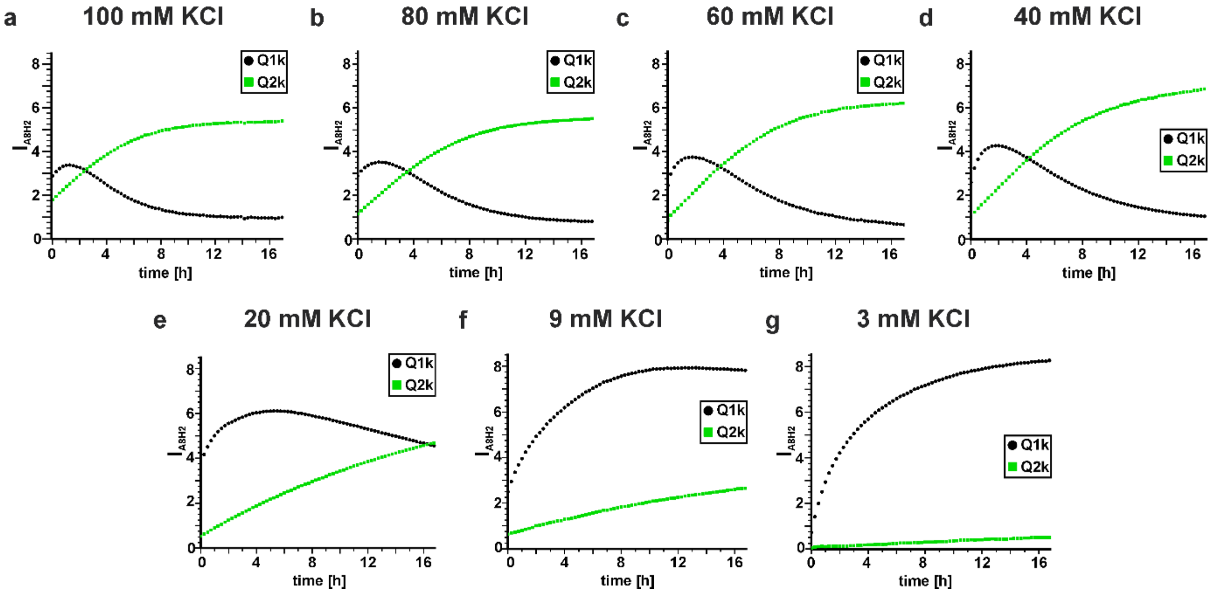

Supplementary Figure 6. The rate of Q1k → Q2k transformation varies with concentration of KCl at 0°C. Plots show Q1k → Q2k transitions at **(a)** 100, **(b)** 80, **(c)** 60, **(d)** 40, **(e)** 20, **(f)** 9 and **(g)** 3 mM KCl concentrations at 0°C by following changes of A8H2 signal integrals in 1D ^{1}H NMR spectra acquired every 15 min over the course of 17h (method 2, Methods, Supplementary Fig. 4). Based on these plots we determined the presence of 60 mM KCl (method 4, Methods) as optimal for structural determination of Q2k, since it is the minimum concentration of KCl where conversion of Q1k → Q2k reaches a plateau after 17h.

1.4 Structural characterization of Q1k

a

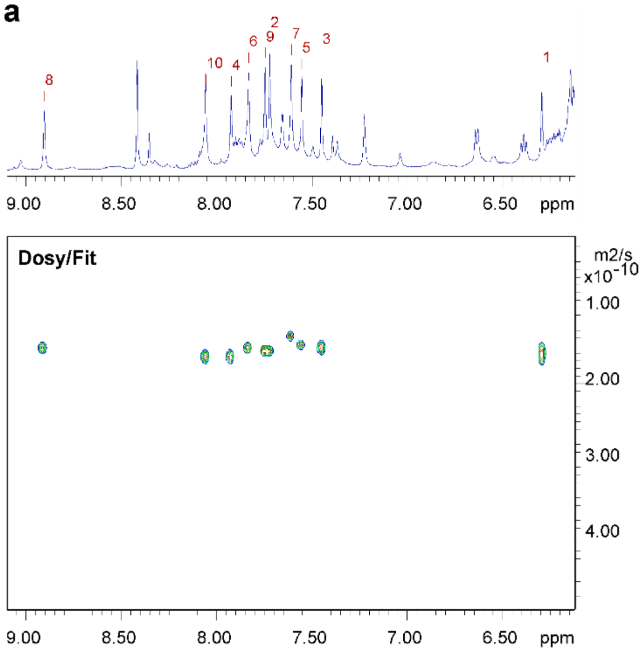

b

Peak name	F2 [ppm]	D_t [m^2/s]	error
1 (G1)	6.296	1.60e-10	3.131e-12
2 (G2)	7.721	1.57e-10	1.305e-12
3 (A3)	7.449	1.52e-10	1.866e-12
4 (G4)	7.924	1.63e-10	1.828e-12
5 (G5)	7.555	1.49e-10	1.015e-12
6 (G6)	7.833	1.52e-10	1.443e-12
7 (G7)	7.610	1.37e-10	9.953e-13
8 (A8)	8.904	1.54e-10	1.683e-12
9 (G9)	7.747	1.58e-10	1.284e-12
10 (G10)	8.057	1.63e-10	1.807e-12

Supplementary Figure 7. NMR diffusion data for Q1k. a H8 region of DOSY NMR spectrum. **b** Translation diffusion coefficients (D_t) of H8 protons of all residues in Q1k. Peak names 1, 2, 3, …, correspond to consecutive residues within d($G_2AG_4AG_2$) as indicated at the beginning of each row. D_t value of Q1k is (1.55 ± 0.016) × 10^{-10} m^2s^{-1}. Spectrum was acquired at 600 MHz, 25°C, 1.0 mM oligonucleotide and 15 mM KCl concentrations in 90% H_2O/10% D_2O (DOSY sample preparation, Methods).

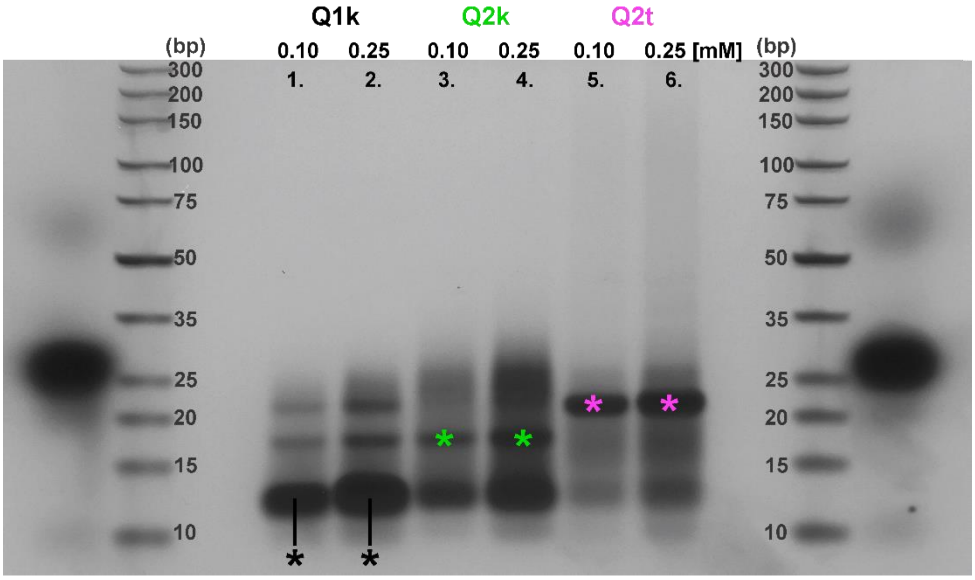

Supplementary Figure 8. Mobility of Q1k, Q2k and Q2t on native PAGE gel (15%). Oligonucleotide concentrations were 0.10 and 0.25 mM per strand (1 and 2.5 nmol) as indicated above individual lane. In the 1st and 2nd lanes we observe strong bands, labeled with black asterisks, between 10 and 15 bp long reference dsDNA, which correspond to Q1k (20 residues). In smaller extend, bands for Q2k (40 residues) and Q2t (40 residues) are also present. In the 3rd and 4th lanes, we see bands corresponding to Q1k, Q2k (labeled with green asterisks) and smearing in place of Q2t bands. In the 5th and 6th lanes, the most intense bands (labeled with pink asterisks) correspond to Q2t, which as expected based on its folding topology displays slightly slower mobility than Q2k and 20 bp long reference dsDNA. Samples were assembled under the conditions where individual (Q1k, Q2k and Q2t) G-quadruplex is dominant in solution (methods 3-5, Methods). Prior to loading, Q1k, Q2k and Q2t samples were diluted with blank solution containing 3, 60 and 15 mM KCl, respectively. As size reference we used Ultra low range DNA ladder (from 300 to 10 bp), where number of base pairs (bp) are indicated next to the individual band. The gel ran at 5°C, 150 V for 2.5h.

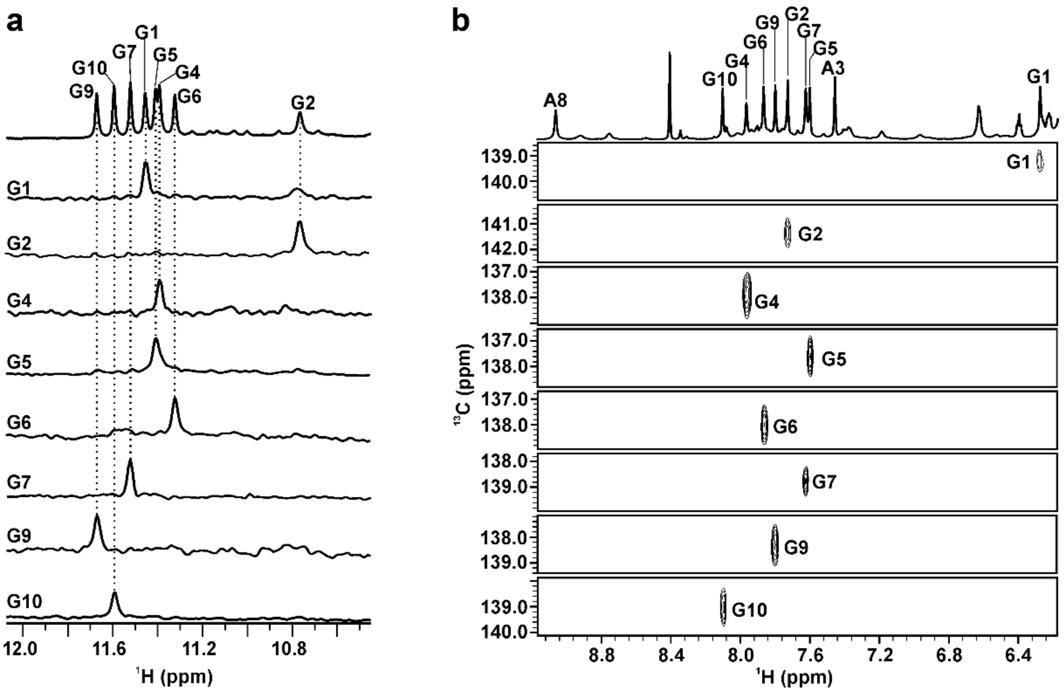

Supplementary Figure 9. G2H1 and G1H8 of Q1k resonate upfield relative to the other H1 and H8 protons, respectively. Unambiguous assignment of **(a)** H1 and **(b)** H8 proton resonances of guanine residues in Q1k. **a** The H1 region of 1D ^{1}H NMR spectrum (top) with a set of 1D ^{15}N-edited HSQC spectra and **(b)** the H8-H1’ region of 1D ^{1}H NMR spectrum (top) with a set of 2D ^{13}C-edited HSQC spectra of Q1k. Spectra were acquired on samples with partially (10%) ^{13}C&^{15}N site-specifically labelled guanine residues, at 600 or 800 MHz, 0°C, 0.6 to 1.4 mM oligonucleotide and 3 mM KCl concentrations in 90% H_2O/10% D_2O. Samples were assembled via method 3 (Methods).

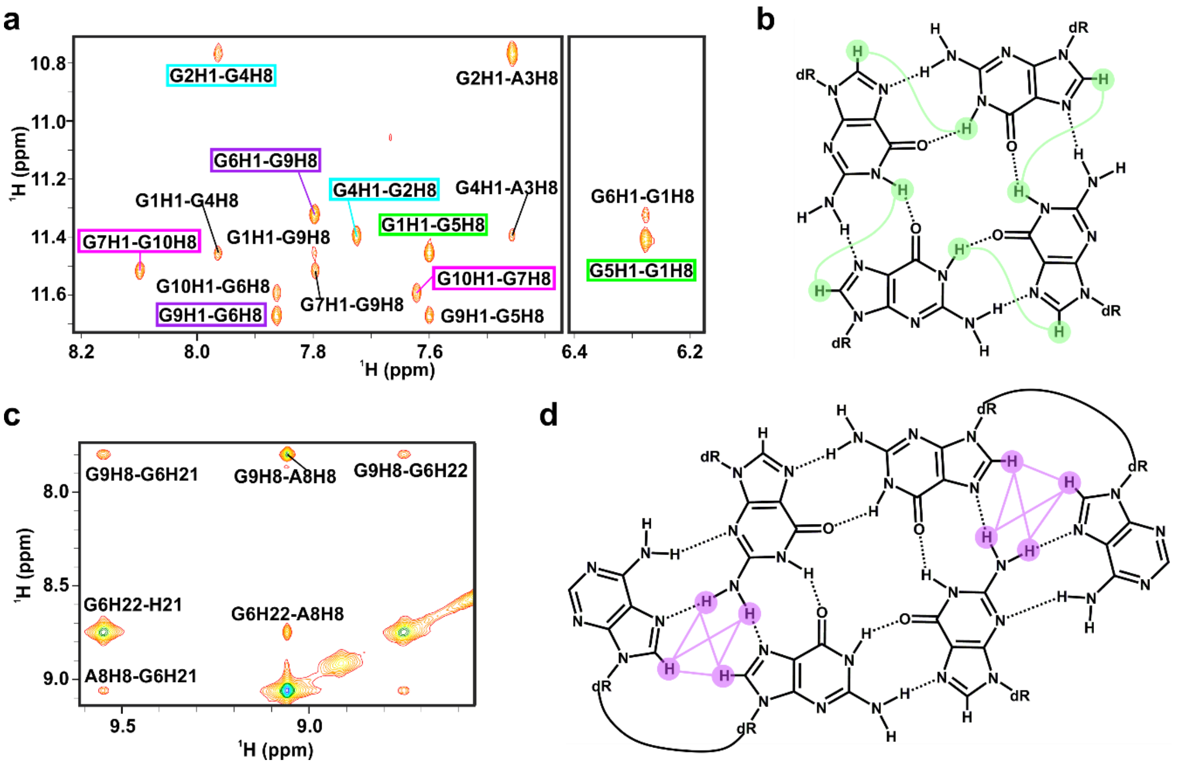

Supplementary Figure 10. Selected regions of 2D NOESY spectrum show NOE cross-peaks crucial for determination of Q1k structure. **a** The H1-H8 region. Green, blue, violet and pink rectangles present pairs of intra-quartet H1-H8 NOEs, which indicate formation of G1-G5-G1-G5, G2-G4-G2-G4, G6-G9-G6-G9 and G7-G10-G7-G10 quartets, respectively. **b** A G-quartet. With green are presented H1-H8 proton connectivities crucial for determination of G-quartet. **c** NOE cross-peaks suggesting formation of A8-(G6-G9-G6-G9)-A8 hexad within Q1k. **d** An A(GGGG)A hexad. With violet are presented proton connectivities crucial for determination of A(GGGG)A hexad. 2D NOESY (τ_m 150 ms) spectrum was acquired at 800 MHz, 0°C, 1.0 mM oligonucleotide and 3 mM KCl concentrations in 90% H_2O/10% D_2O. Sample was assembled via method 3 (Methods).

1.5 Structural characterization of Q2k

a

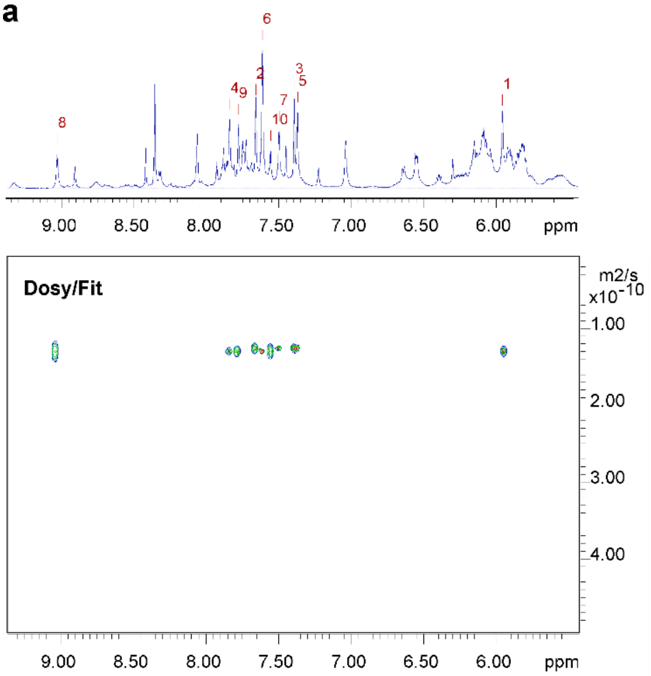

b

Peak name	F2 [ppm]	D_t [m^2/s]	error
1 (G1)	5.952	1.29e-10	1.156e-12
2 (G2)	7.657	1.27e-10	1.276e-12
3 (A3)	7.391	1.25e-10	1.024e-12
4 (G4)	7.837	1.30e-10	9.596e-13
5 (G5)	7.368	1.25e-10	8.551e-13
6 (G6)	7.612	1.29e-10	5.011e-13
7 (G7)	7.496	1.24e-10	6.735e-13
8 (A8)	9.029	1.31e-10	3.204e-12
9 (G9)	7.778	1.30e-10	1.693e-12
10 (G10)	7.554	1.30e-10	2.329e-12

Supplementary Figure 11. NMR diffusion data for Q2k. **a** H8 region of DOSY NMR spectrum of Q2k. **b** D_t of H8 protons of all residues in Q2k. Peak names 1, 2, 3, …, correspond to consecutive residues within d($G_2AG_4AG_2$) as indicated at the beginning of each row. D_t value for Q2k is (1.28 ± 0.014) × 10^{-10} m^2s^{-1}. Spectrum was acquired at 600 MHz, 25°C, 1.0 mM oligonucleotide and 15 mM KCl concentrations in 90% H_2O/10% D_2O (Methods).

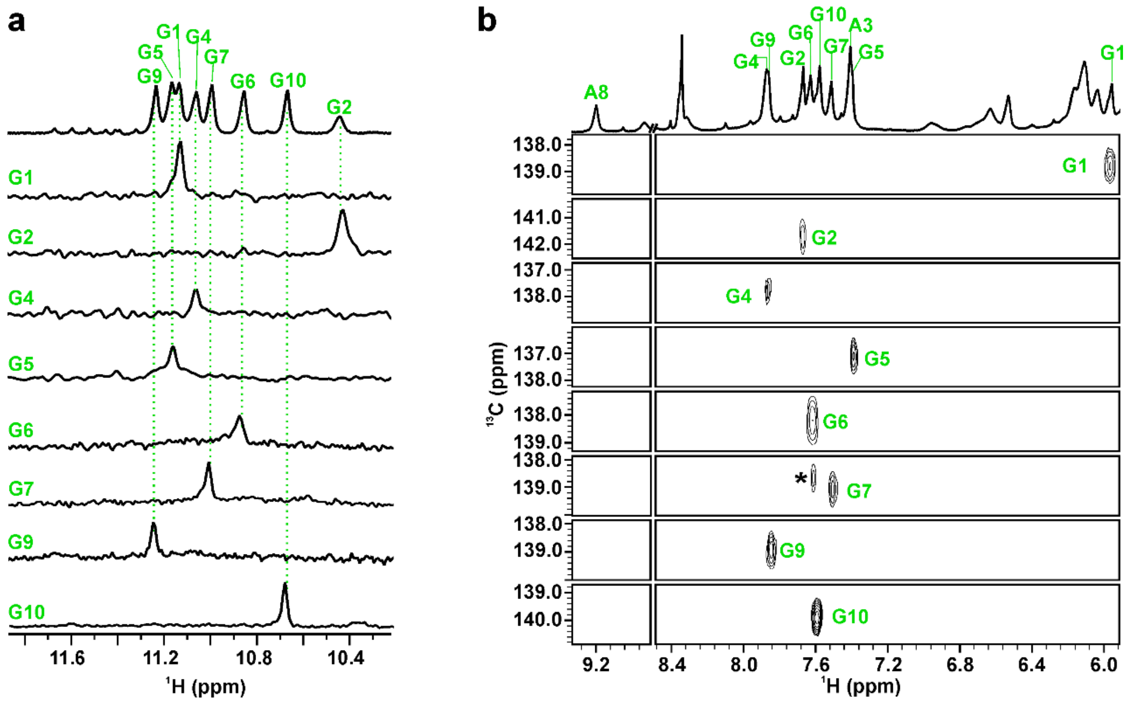

Supplementary Figure 12. Similar as in Q1k, G2H1 and G1H8 resonate upfield relative to the other H1 and H8 protons in Q2k, respectively. Unambiguous assignment of **(a)** H1 and **(b)** H8 proton resonances of guanine residues in Q2k. **a** The H1 region of 1D ^{1}H NMR spectrum (top) with a set of 1D ^{15}N-edited HSQC spectra and **(b)** the H8-H1' region of 1D ^{1}H NMR spectrum (top) with a set of 2D ^{13}C-edited HSQC spectra of Q2k. Black asterisk in **(b)** presents G7H8 signal corresponding to Q1k. Spectra were acquired on samples with partially (10%) ^{13}C&^{15}N site-specifically labelled guanine residues, at 600 or 800 MHz, 0°C, 0.6 to 1.3 mM oligonucleotide and 60 mM KCl concentrations in 90% H_2O/10% D_2O. Samples were assembled via method 4 (Methods).

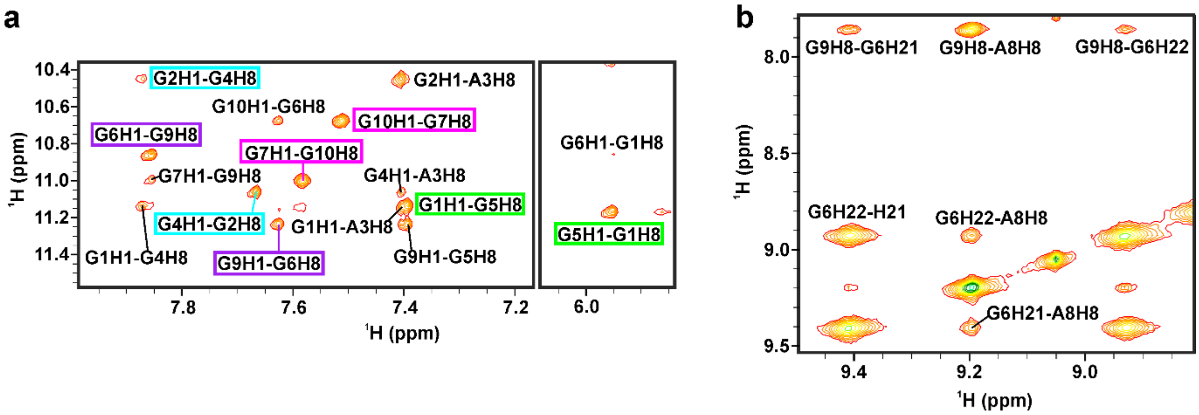

Supplementary Figure 13. Selected regions of 2D NOESY spectrum show NOE cross-peaks, which indicate that Q2k exhibits the same structural elements as Q1k. **a** The H1-H8 region. Green, blue, violet and pink rectangles present pairs of intra-quartet H1-H8 NOEs, which determine formation of G1-G5-G1-G5, G2-G4-G2-G4, G6-G9-G6-G9 and G7-G10-G7-G10 quartets, respectively. **b** Cross-peaks characteristic for A(GGGG)A hexad formation. 2D NOESY (τ_m 150 ms) spectrum was acquired at 800 MHz, 0°C, 1.0 mM oligonucleotide and 60 mM KCl concentrations in 90% H_2O/10% D_2O. Sample was assembled via method 4 (Methods).

1.6 Identification and characterization of Q2t

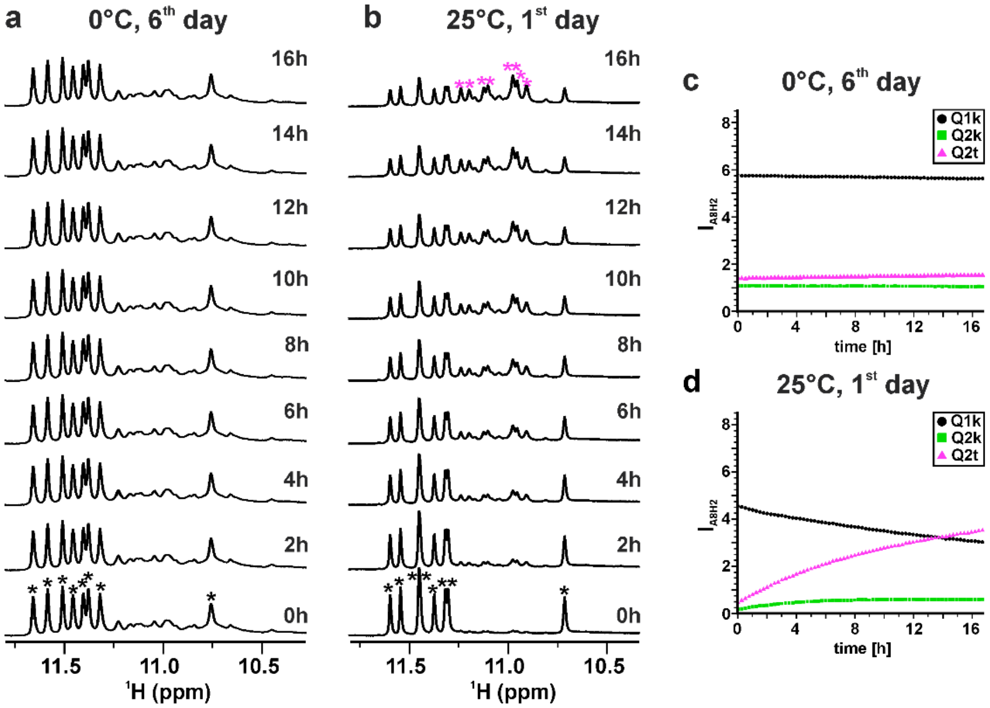

Supplementary Figure 14. Higher temperature shifts equilibrium towards formation of Q2t. H1 regions of a set of 1D ^{1}H NMR spectra acquired in 3 mM KCl **(a)** on the 6th day at 0°C and **(b)** on the 1st day at 25°C after the addition of KCl. **a,b** Black and pink asterisks denote H1 signals, which correspond to Q1k and Q2t, respectively. We observe signals corresponding to Q2t **(a)** on the 6th day after the addition of KCl to 3 mM concentration at 0°C (method 2, Methods). In order to increase the rate of Q2t formation, we acquired **(b)** 1D ^{1}H NMR experiments at 25°C on freshly prepared sample with the same solution conditions (method 2, Methods). Plots show Q1k→Q2k→Q2t transitions where changes of A8H2 signal integrals of individual structure are presented as a function of time at **(c)** 0°C and **(d)** 25°C. Signal integrals were obtained from a set of 1D ^{1}H NMR spectra in **(a,b)**. **d** Plot shows that formation of Q2t is dominant process at 25° at given conditions. We used these plots in order to identify rate determining step of d($G_2AG_4AG_2$) G-wire self-assembly. At (**c**) 0°C, system does not have enough energy for an immediate structural transition of Q2k to Q2t, therefore formation of Q2t determines rate of self-assembly. In contrast, at (**d**) 25°C Q2t is formed from Q2k rather quickly, therefore the formation of Q2k is rate-determining step of d($G_2AG_4AG_2$) self-assembly at 25°C and higher temperatures. Spectra were acquired every 15 min for 17h (PAD experiments, Methods). For clarity, only selected spectra are shown. Spectra in **(a,b)** were acquired at 800 MHz, 1.0 mM oligonucleotide and 3 mM KCl concentrations in 90% H_2O/10% D_2O.

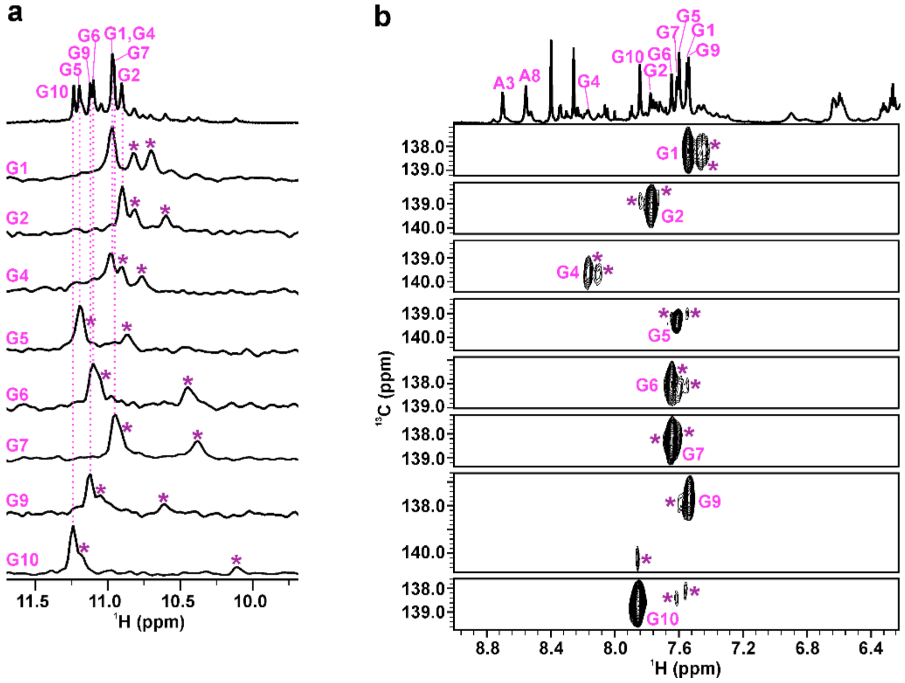

Supplementary Figure 15. Q2t is structurally different as indicated by G2H1 and G1H8, which resonate within same chemical shift range as the other H1 and H8 protons. Unambiguous assignment of **(a)** H1 and **(b)** H8 proton resonances of guanine residues in Q2t and Q4t. **a** The H1 region of 1D ^{1}H NMR spectrum (top) with a set of 1D ^{15}N-edited HSQC spectra and **(b)** the H8-H1' region of 1D ^{1}H NMR spectrum (top) with a set of 2D ^{13}C-edited HSQC spectra of Q2t and Q4t. Violet asterisks mark resonances corresponding to protons of guanines in Q4t. Spectra were acquired on samples with partially (10%) ^{13}C&^{15}N site-specifically labelled guanine residues, at 600 MHz, 25°C, up to 1.0 mM oligonucleotide and up to 20 mM KCl concentrations in 90% H_2O/10% D_2O. Samples were assembled via method 5 (Methods).

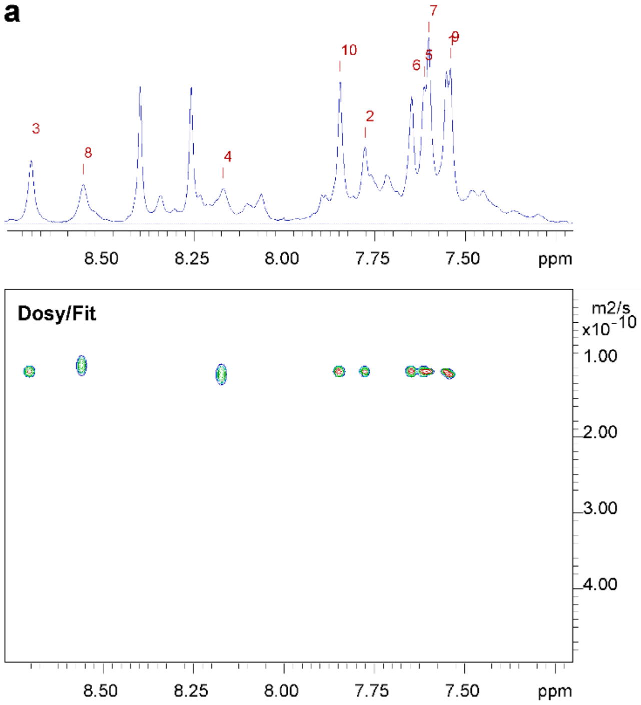

b

Peak name	F2 [ppm]	D_t [m^2/s]	error
1 (G1)	7.551	1.15e-10	6.344e-13
2 (G2)	7.777	1.15e-10	1.097e-12
3 (A3)	8.702	1.14e-10	1.441e-12
4 (G4)	8.170	1.16e-10	2.764e-12
5 (G5)	7.613	1.12e-10	1.010e-12
6 (G6)	7.647	1.14e-10	1.247e-12
7 (G7)	7.600	1.14e-10	5.385e-13
8 (A8)	8.557	1.05e-10	2.661e-12
9 (G9)	7.541	1.16e-10	7.352e-13
10 (G10)	7.847	1.15e-10	1.175e-12

Supplementary Figure 16. NMR diffusion data for Q2t. **a** H8 region of DOSY NMR spectrum. **b** D_t of H8 protons of all residues in Q2t. Peak names 1, 2, 3, …, correspond to consecutive residues within d($G_2AG_4AG_2$) as indicated at the beginning of each row. D_t value of Q2t is (1.14 ± 0.013) × 10^{-10} m^2s^{-1}. Spectrum was acquired at 600 MHz, 25°C, 1.0 mM oligonucleotide and 15 mM KCl concentrations in 90% H_2O/10% D_2O (DOSY sample preparation, Methods).

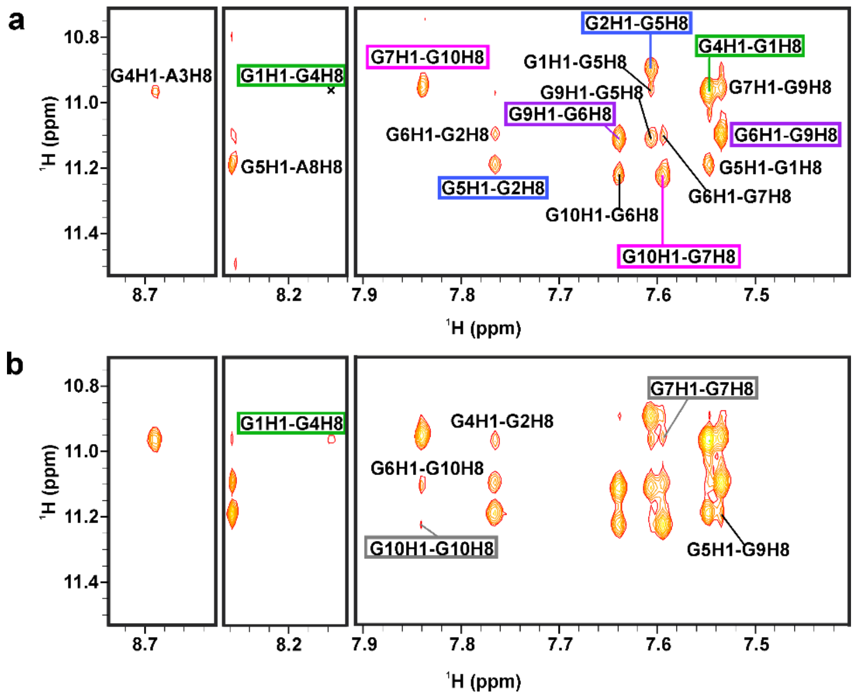

Supplementary Figure 17. Q2t is an all-parallel G-quadruplex. **a** The H1-H8 regions of 2D NOESY spectra with τ_m of 150 ms and **(b)** τ_m of 250 ms mixing times. **a** Violet and pink rectangles present pairs of intra-quartet H1-H8 NOEs, which confirm that G6-G9-G6-G9 and G7-G10-G7-G10 quartets are preserved. Green and blue rectangles present pairs of intra-quartet H1-H8 NOEs, which support structural rearrangement and formation of G1-G4-G1-G4 and G2-G5-G2-G5 quartets, respectively. Cross in **(a)** denotes weak G1H1-G4H8 NOE. **b** Grey rectangles present inter-quadruplex G7H1-G7H8 and G10H1-G10H8 NOEs, which support 3'-3' stacking within Q2t. Spectra were acquired at 800 MHz, 25°C, 1.0 mM oligonucleotide and 15 mM KCl concentrations in 90% H_2O/10% D_2O. Sample was assembled via method 5 (Methods).

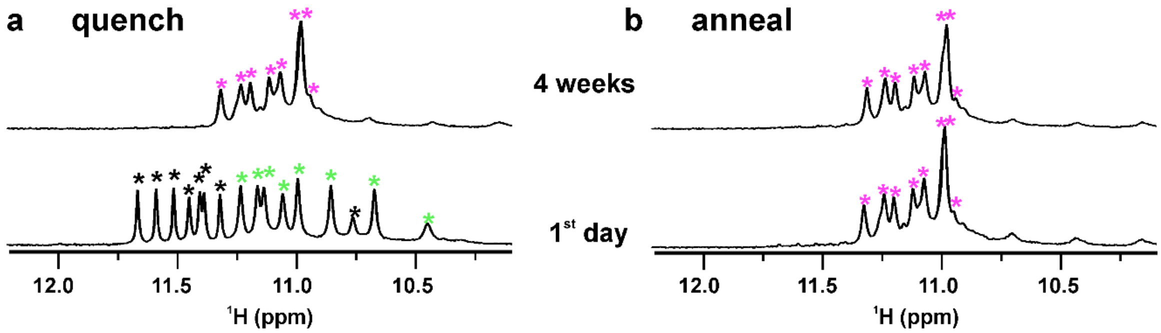

Supplementary Figure 18. Qk-type of G-quadruplexes is kinetically preferred, whereas Qt-type is thermodynamically favored. H1 regions of 1D ^{1}H NMR spectra of **(a)** quenched and **(b)** annealed d($G_2AG_4AG_2$) in 15 mM KCl. **a,b** 1D ^{1}H NMR spectra were acquired within 1st day (bottom spectra) and 4 weeks after (top spectra) the heat treatment. **a** Two sets of signals are observed in the bottom spectrum, which correspond to H1 protons of Q1k (black asterisks) and Q2k (green asterisks). After 4 weeks (top spectrum) we observe only signals corresponding to Q2t (pink asterisks). **b** Only Q2t is present in annealed sample, where no changes are observed in 1D ^{1}H NMR spectra with time. Spectra were acquired at 800 MHz, 0°C, 1.0 mM oligonucleotide and 15 mM KCl concentrations in 90% H_2O/10% D_2O.

1.7 Characterization of Q4t

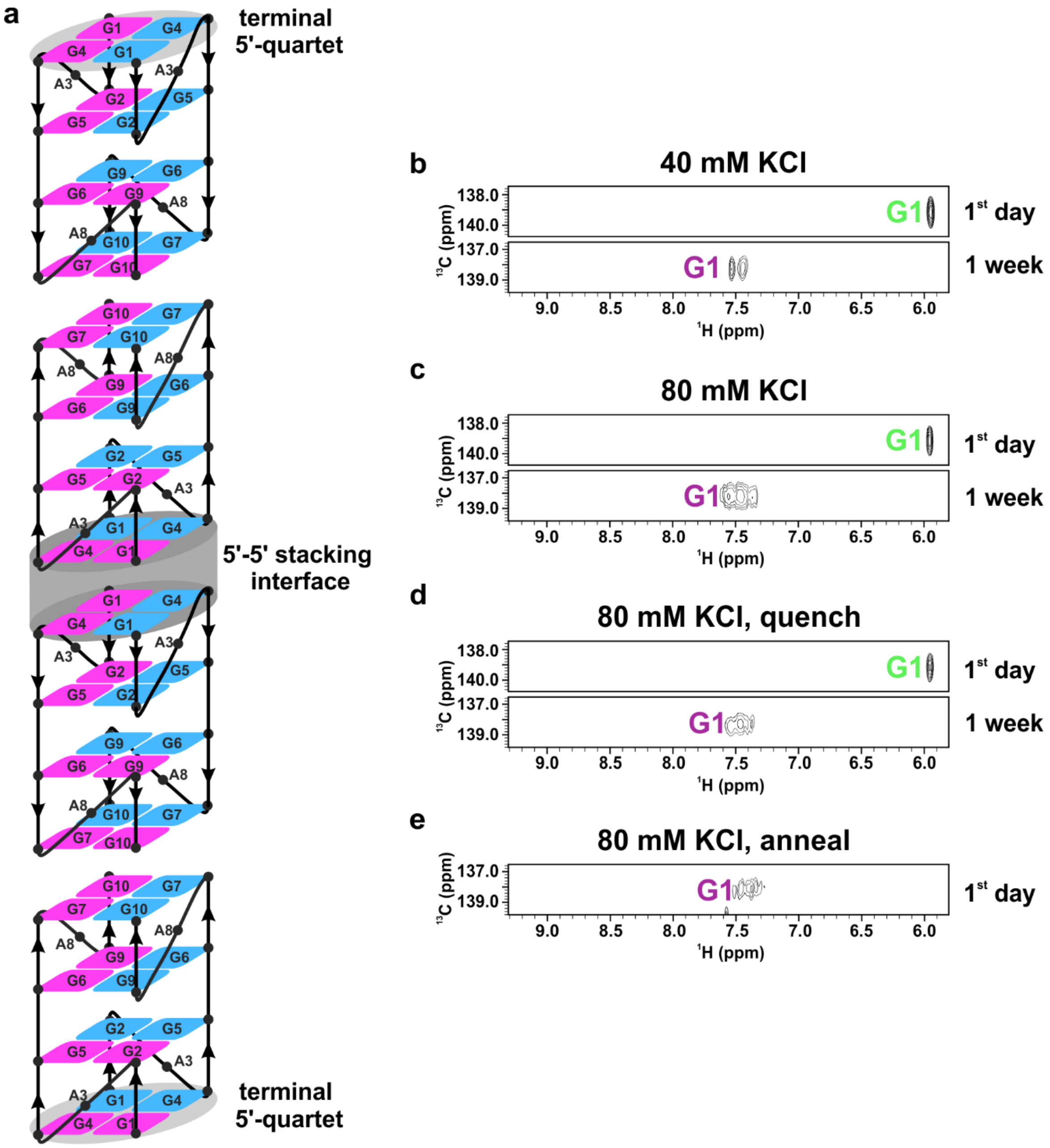

Supplementary Figure 19. All d($G_2AG_4AG_2$) G-wires are Qt-type of structures. **a** Folding topology of Q4t. Terminal 5'-quartets and 5'-5' stacking interface are emphasized with light and dark grey ellipses, respectively. 2D ^{13}C-edited HSQC spectra of d($G_2AG_4AG_2$) with 10% isotopically enriched G1 residue in **(b)** 40 and **(c-e)** 80 mM KCl concentrations. Spectra were acquired on the 1st day and after 1 week following the addition of KCl as indicated on the right site of spectra. Samples were prepared **(b,c)** without heat treatment or with **(d)** quenching and **(e)** annealing as indicated above spectra. Green and pink colors mark cross-peaks that correspond to Q2k and Qt-type of structures, respectively. Spectra were acquired at 600 MHz, 25°C, 1.0 mM oligonucleotide concentration in 90% H_2O/10% D_2O.

1.8 Phase diagram of $d(G_2AG_4AG_2)$ self-assembly

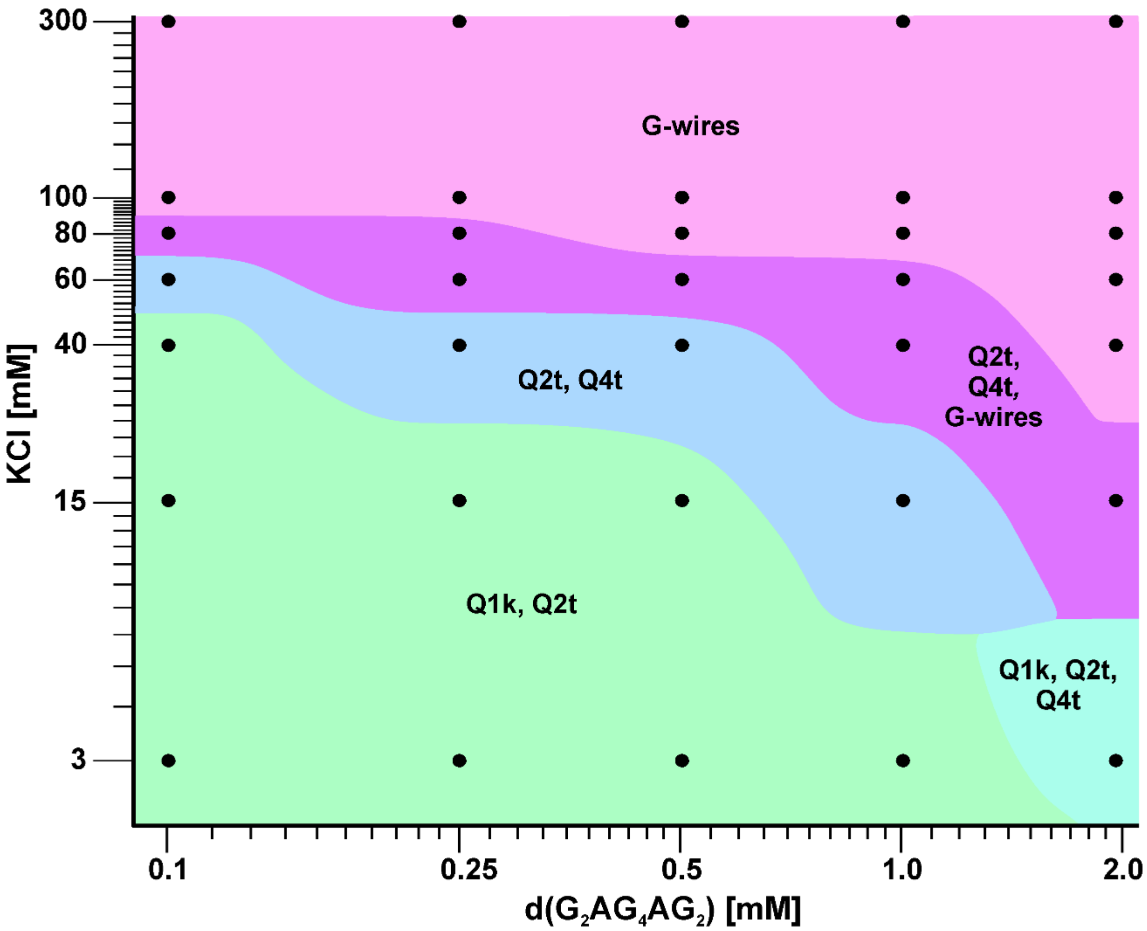

Supplementary Figure 20. Simplified phase diagram showing how oligonucleotide and K^+ concentration influence G-wire self-assembly. At conditions represented with black dots, we have acquired 1D ^1H NMR spectra in order to obtain information about structures present in solution at 25°C and equilibrium state. Sample preparation is described in Methods (Phase diagram sample preparation).

1.9 Characterization of G-wires with thymines and cytosines in loops

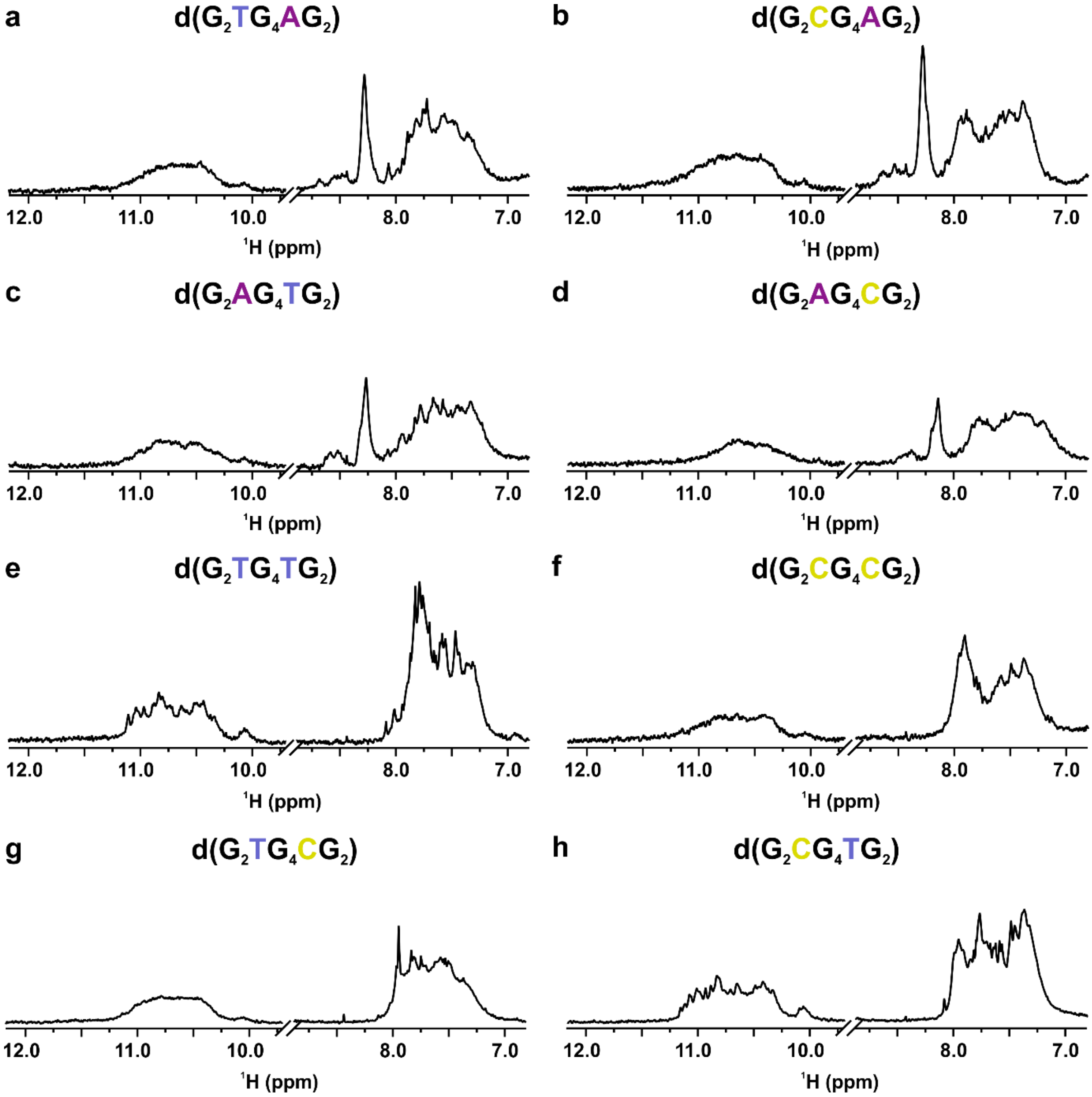

Supplementary Figure 21. All modified oligonucleotides form G-wires. H1 and H8 regions of 1D ^{1}H NMR spectra of G-wires formed by **(a)** $d(G_2\underline{T}G_4AG_2)$, **(b)** $d(G_2\underline{C}G_4AG_2)$, **(c)** $d(G_2AG_4\underline{T}G_2)$, **(d)** $d(G_2AG_4\underline{C}G_2)$, **(e)** $d(G_2\underline{T}G_4\underline{T}G_2)$, **(f)** $d(G_2\underline{C}G_4\underline{C}G_2)$, **(g)** $d(G_2\underline{T}G_4\underline{C}G_2)$ and **(h)** $d(G_2\underline{C}G_4\underline{T}G_2)$. Spectra were acquired at 600 and 800 MHz, 25°C, 1.0 mM oligonucleotide and 100 mM KCl concentrations in 90% H_2O/10% D_2O. Samples were assembled via method 1 (Methods).

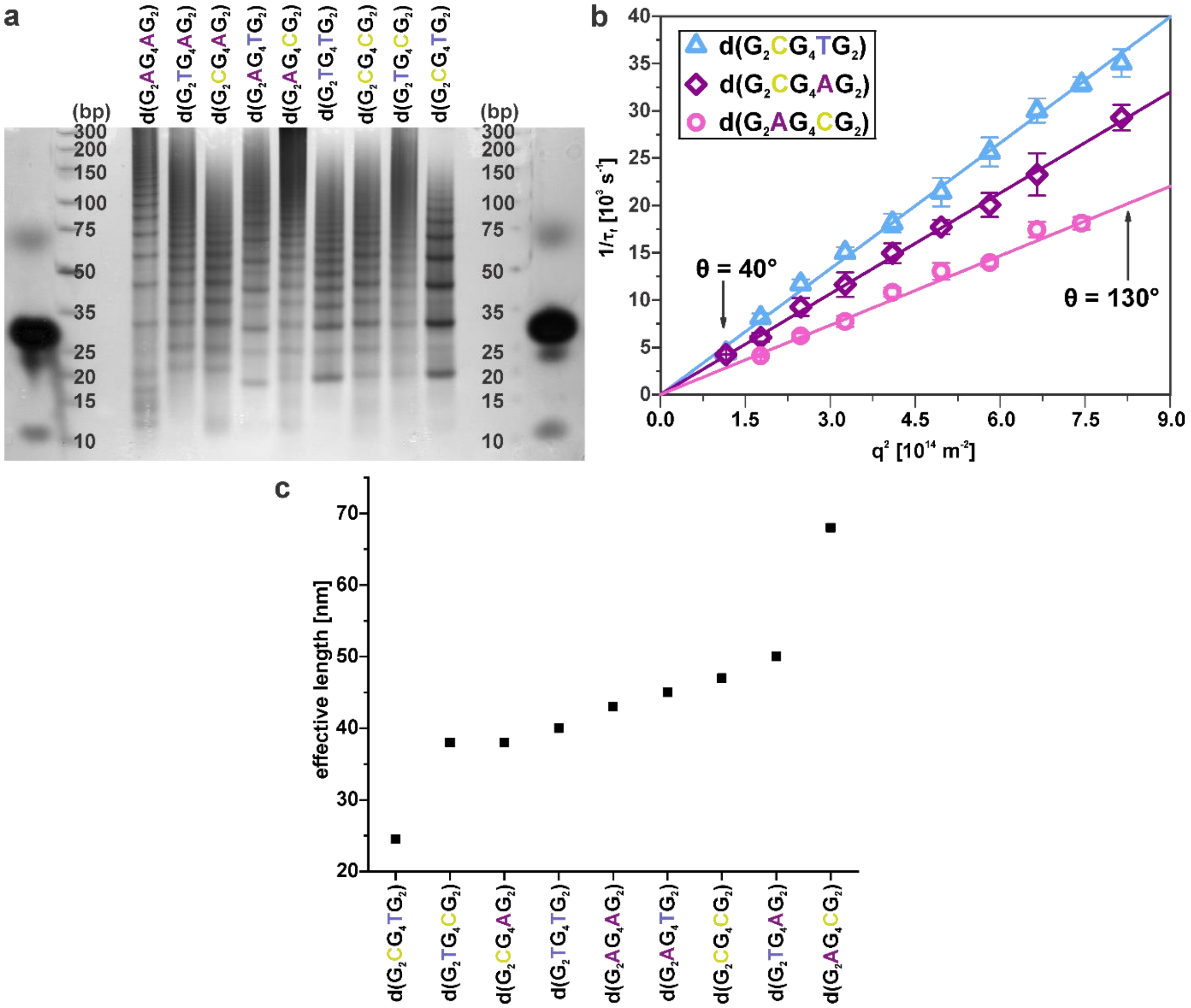

Supplementary Figure 22. Length of G-wires can be controlled by substitutions of residues in loops. **a** Native PAGE (15%) gel shows ladder pattern with smearing for all G-wires. In agreement with D_t values, we see the most pronounced smearing for the longest $d(G_2AG_4\underline{C}G_2)$ and least pronounced smearing for the shortest $d(G_2\underline{C}G_4\underline{T}G_2)$ among G-wires with loop residue substitutions. In the native PAGE gel we can nicely see bands that correspond to Q2t (above 20 bp), Q4t (between 35 and 50 bp), Q8t (between 75 and 100 bp) and longer Qt structures, which are in accordance with the mechanism of $d(G_2AG_4AG_2)$ self-assembly, where G-wire growth occurs via 5'-5' stacking of Q2t (or Q4t) structures. Interestingly, we also observe additional bands, e.g., potentially corresponding to Q3t (between 25 and 35 bp) and Q5t (between 50 and 75 bp). However, formation of such structures, e.g., Q3t and Q5t would require presence of Q1t, which we did not observe by NMR spectroscopy. Therefore, the appearance of additional bands in the native PAGE gel might be explained by formation of hybrid structures, where Q1k stacks on Qnt structures. One must note, that we did not observe such Qk-Qt hybrid structures in solution as demonstrated by 2D ^{13}C-filtered HSQC experiments on $d(G_2AG_4AG_2)$ sample with partially isotopically labeled G1 residues (Supplementary Fig. 19b-e). Oligonucleotide concentrations were 0.25 mM (2.5 nmol) and their sequences are presented above individual lane. 25 mM KCl was added to the gel and running buffer for stabilization of structures. As standard for size we used Thermo Scientific GeneRuler Ultra Low Range DNA Ladder (from 300 to 10 bp), as seen on left and right side of the gel. **b** DLS measurements for three of the modified oligonucleotides. From experimental autocorrelation curves we obtained the characteristic relaxation times of the diffusive processes in the solution. The faster of the two processes is associated with G-quadruplex diffusion. The square of the scattering vector q and the inverse relaxation time have a linear dependence (Methods) and the slope represents the diffusion coefficient. The values obtained from the above graph are: $D_t = 0.443 \pm 0.007 \times 10^{-10}$ m^{2}s^{-1} for $d(G_2\underline{C}G_4\underline{T}G_2)$, $D_t = 0.355 \pm 0.003 \times 10^{-10}$ m^{2}s^{-1} for $d(G_2\underline{C}G_4AG_2)$ and $D_t = 0.245 \pm 0.004 \times 10^{-10}$ m^{2}s^{-1} for $d(G_2AG_4\underline{C}G_2)$. Samples were assembled via method 1 (Methods). **c** In plot, modified oligonucleotides are arranged in order of increasing effective length of resulting G-wires (from left to right) obtained from DLS measurements.

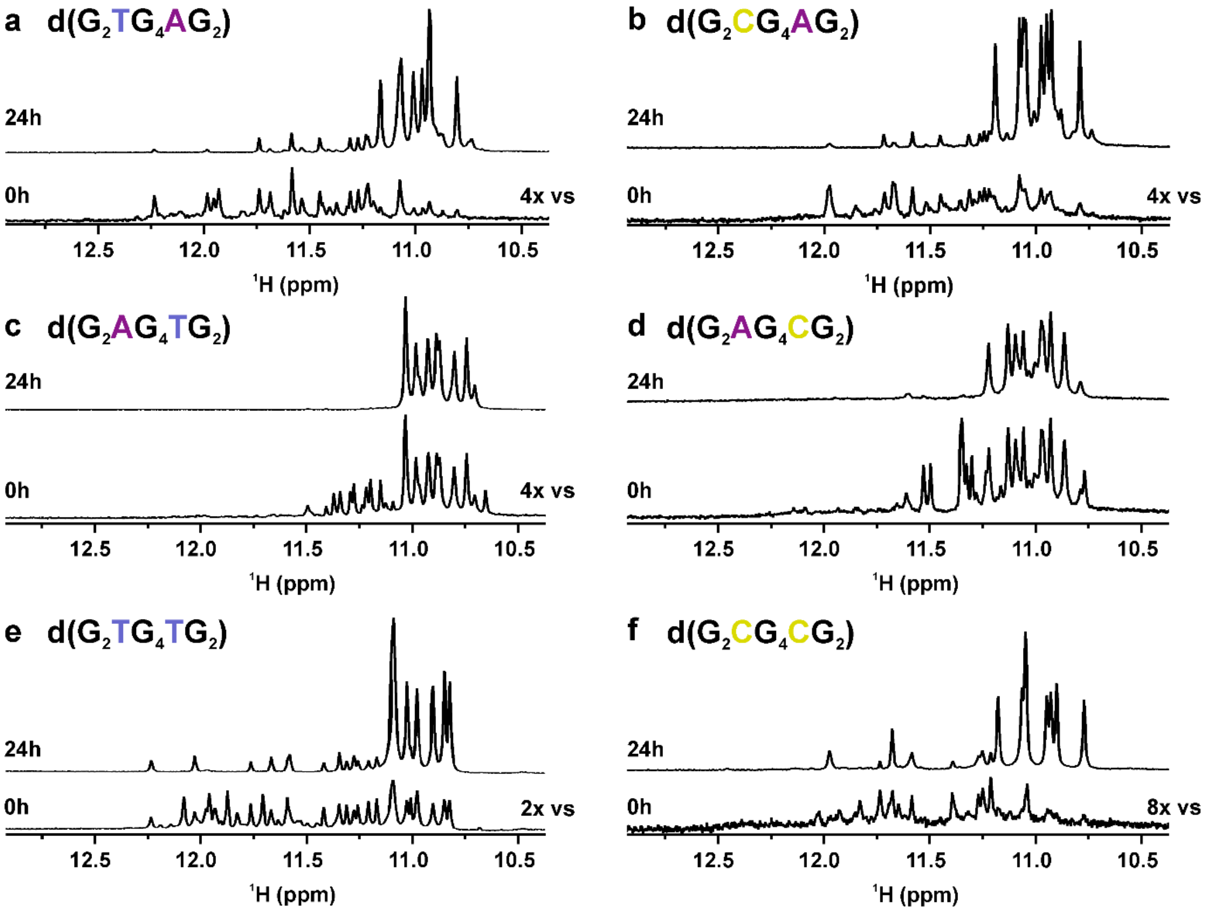

Supplementary Figure 23. H1 regions of 1D ^{1}H NMR spectra of **(a)** d($G_2\underline{T}G_4AG_2$), **(b)** d($G_2\underline{C}G_4AG_2$), **(c)** d($G_2AG_4\underline{T}G_2$), **(d)** d($G_2AG_4\underline{C}G_2$), **(e)** d($G_2\underline{T}G_4\underline{T}G_2$) and **(f)** d($G_2\underline{C}G_4\underline{C}G_2$) acquired immediately (bottom spectra) after addition of KCl to final 3 mM concentration in solution and 1 day later (top spectra). Spectra were acquired at 800 MHz, 25°C, 1.0 mM oligonucleotide and 3 mM KCl concentrations in 90% H_2O/10% D_2O.

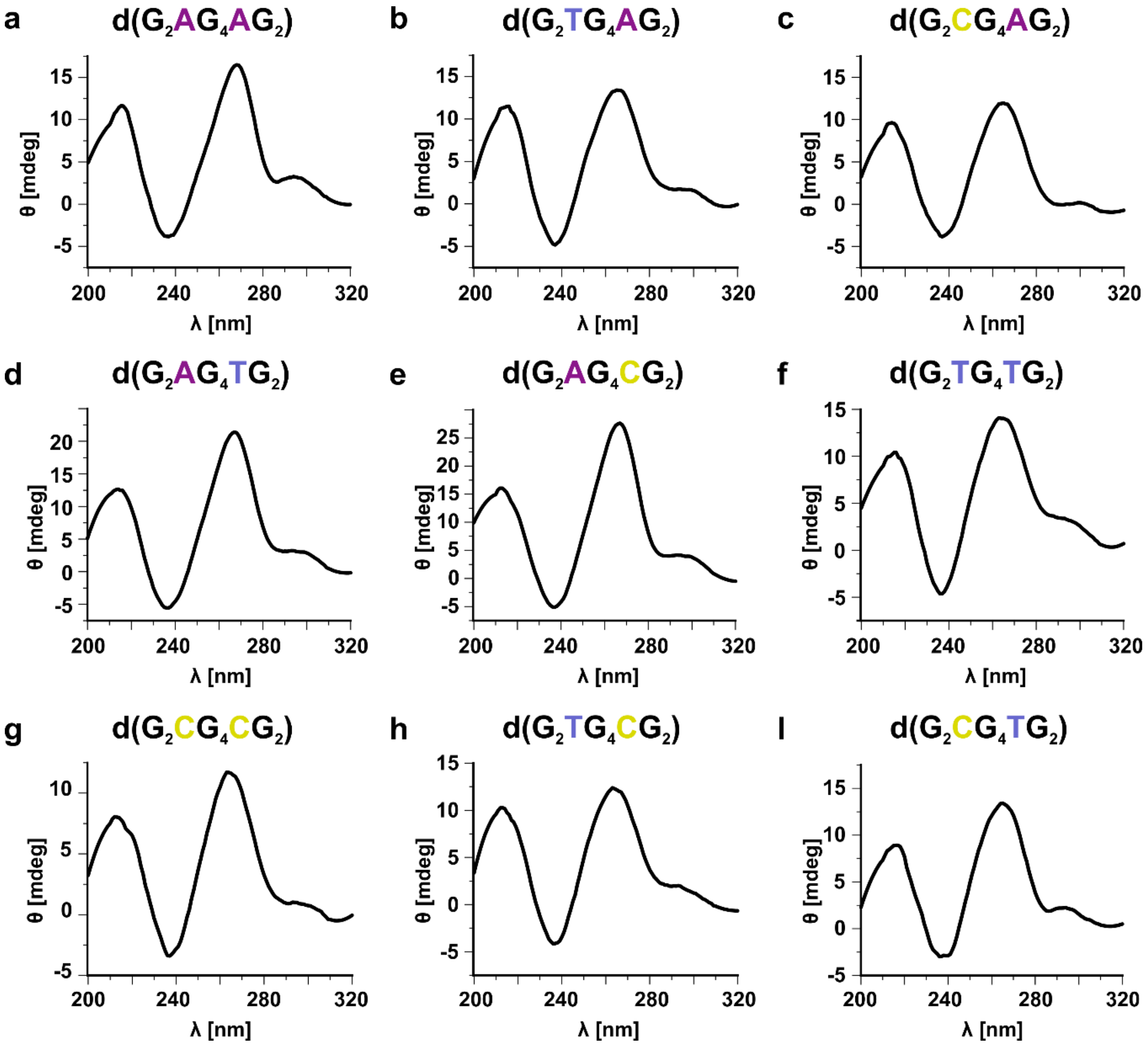

Supplementary Figure 24. G-wires formed by parent and modified oligonucleotides exhibit similar CD profiles. CD spectra of **(a)** d(G$_2$AG$_4$AG$_2$), **(b)** d(G$_2$TG$_4$AG$_2$), **(c)** d(G$_2$CG$_4$AG$_2$), **(d)** d(G$_2$AG$_4$TG$_2$), **(e)** d(G$_2$AG$_4$CG$_2$), **(f)** d(G$_2$TG$_4$TG$_2$), **(g)** d(G$_2$CG$_4$CG$_2$), **(h)** d(G$_2$TG$_4$CG$_2$), **(i)** d(G$_2$CG$_4$TG$_2$) G-wires. Similar CD profiles indicate similar structural type of G-wires. Spectra were acquired at 25°C, 1.0 mM oligonucleotide and 100 mM KCl concentrations in 90% H_2O/10% D_2O. Samples were assembled via method 1 (Methods).

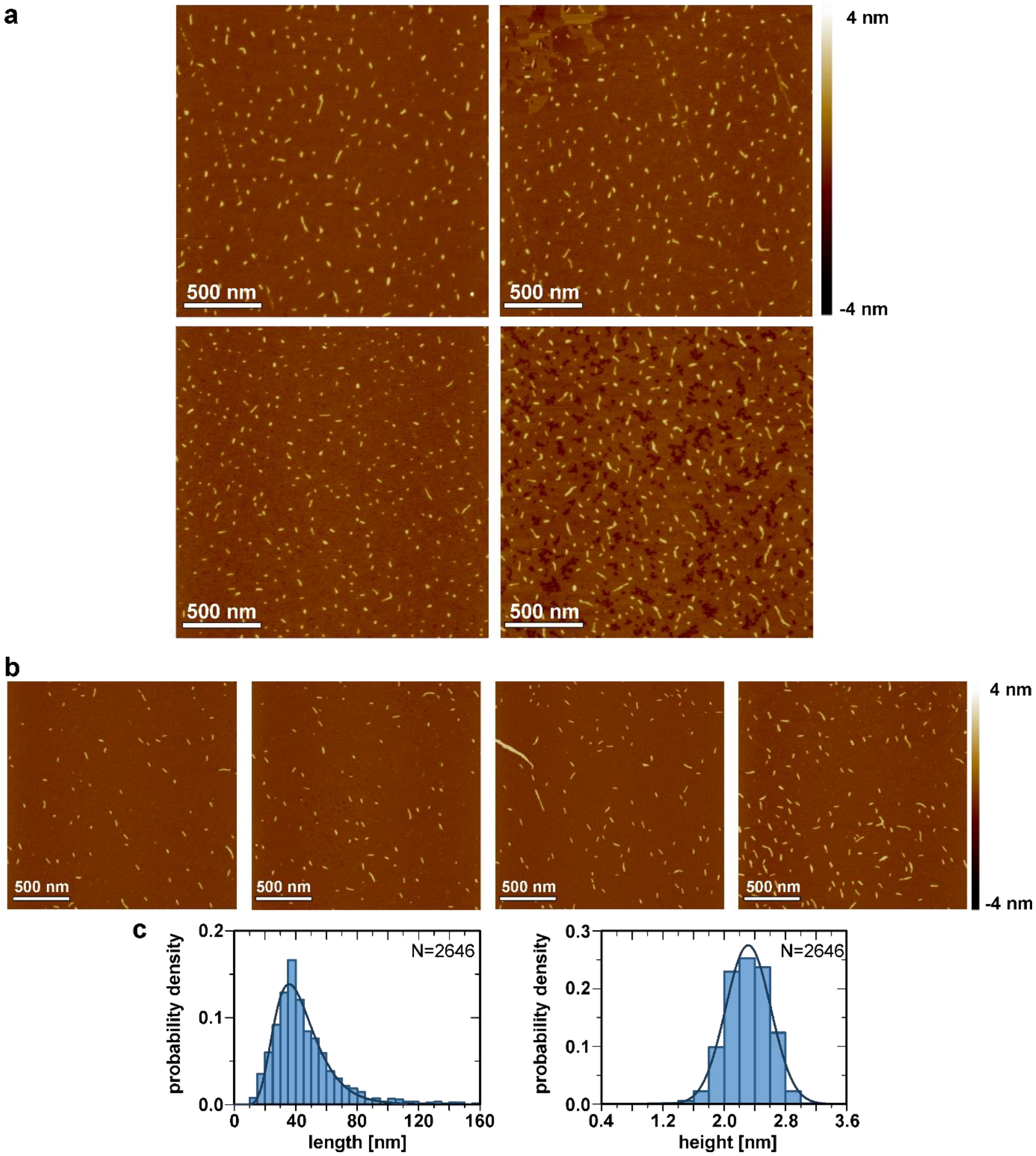

Supplementary Figure 25. AFM images of d($G_2AG_4\underline{C}G_2$) G-wires. Samples were deposited on muscovite mica **(a)** pre-treated with saturated solution of $MgCl_2$ and **(b)** not pre-treated with $MgCl_2$. Sample deposition concentration was 0.2 µM, which enable visualization of individual G-wires. **c** Length and height histograms correspond to sample deposited on not pre-treated muscovite mica in **(b)** where statistics were collected over 20 images. Sample was assembled via method 1 (Methods).

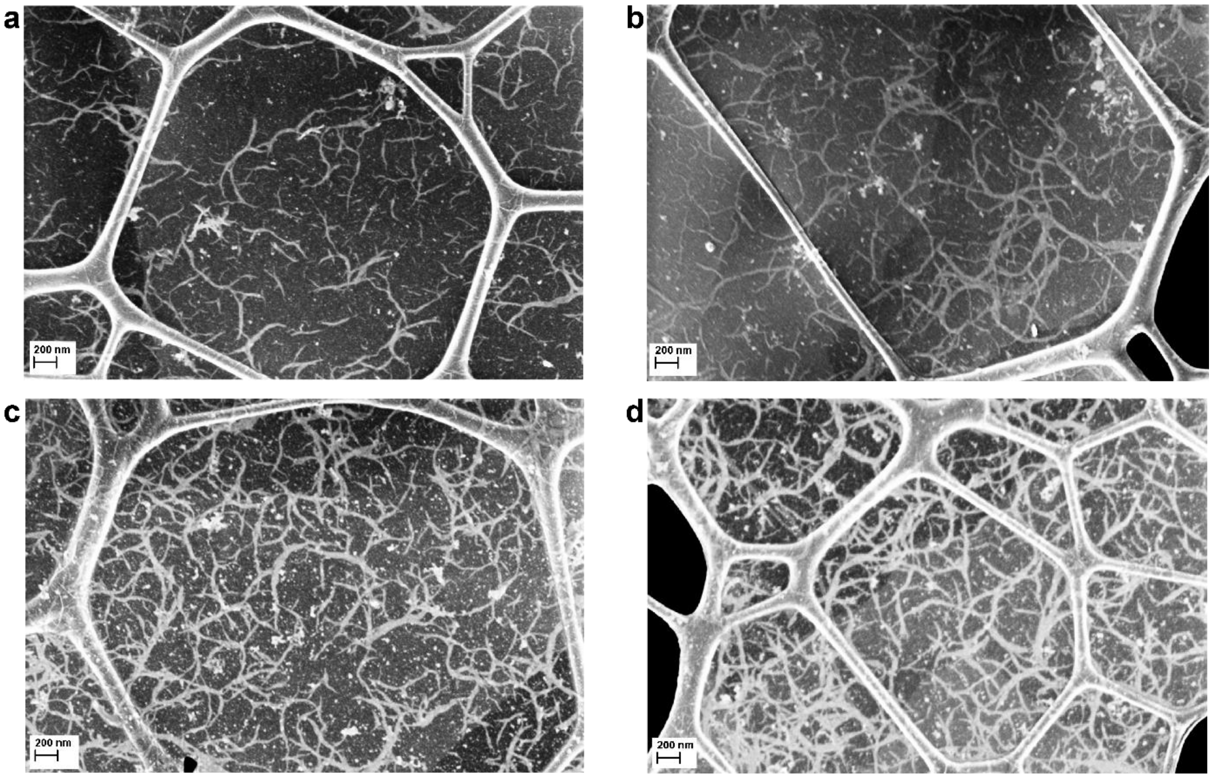

Supplementary Figure 26. SEM images of d($G_2AG_4\underline{C}G_2$) G-wires. Spots where we observe **(a,b)** more individual and **(c,d)** bigger deposits of G-wires. Sample deposition concentration was 1.0 mM. Sample was assembled via method 1 (Methods).

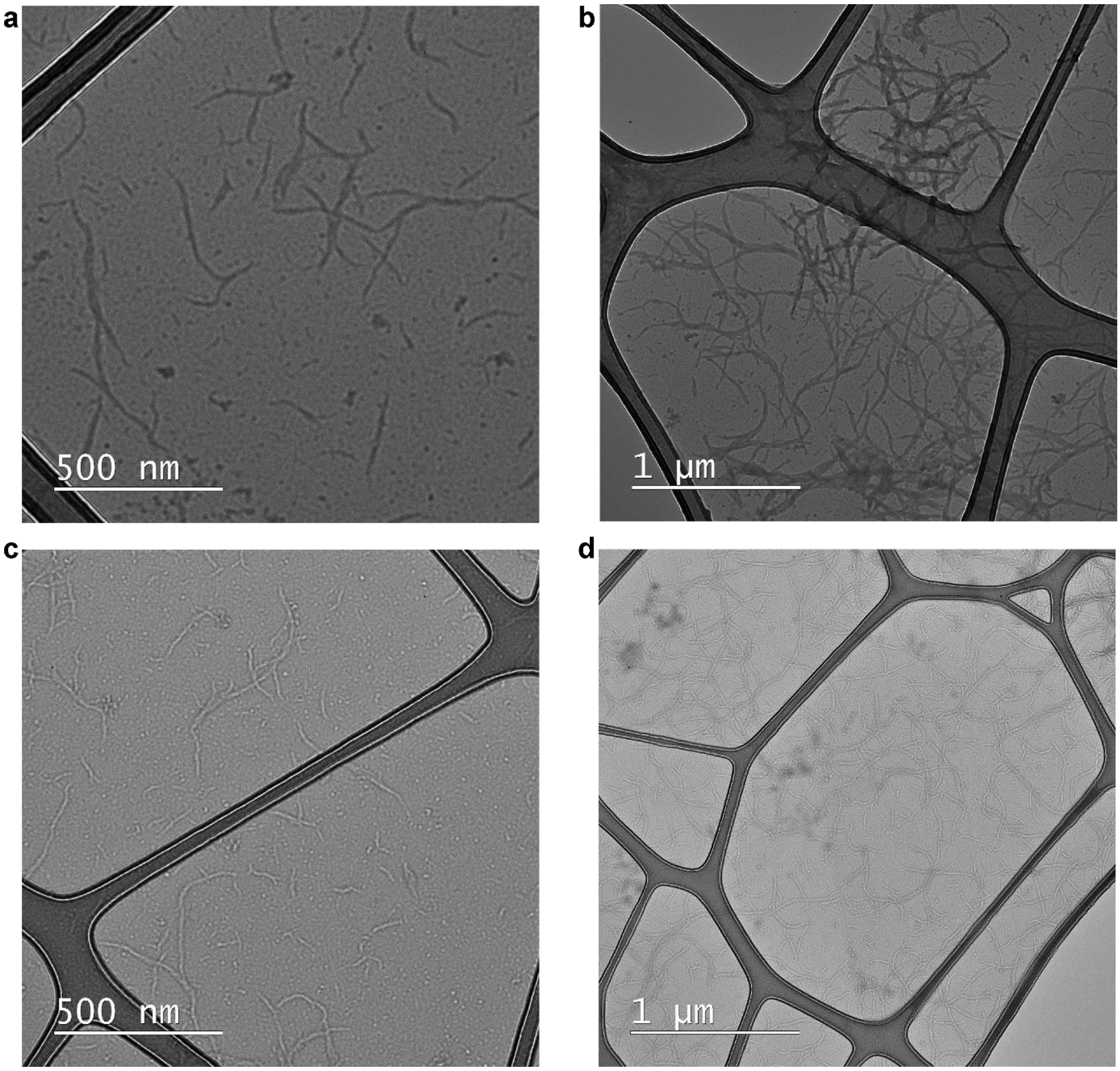

Supplementary Figure 27. Conventional TEM images of unstained d($G_2AG_4\underline{C}G_2$) G-wires. **a,b** TEM images were taken at substantial under-focus and **(c,d)** over-focus. Spots where we observed **(a,c)** more individual and **(b,d)** bigger deposits of G-wires. Sample deposition concentration was 1.0 mM. Sample was assembled via method 1 (Methods).

1.10 G-wires thermal stability studies

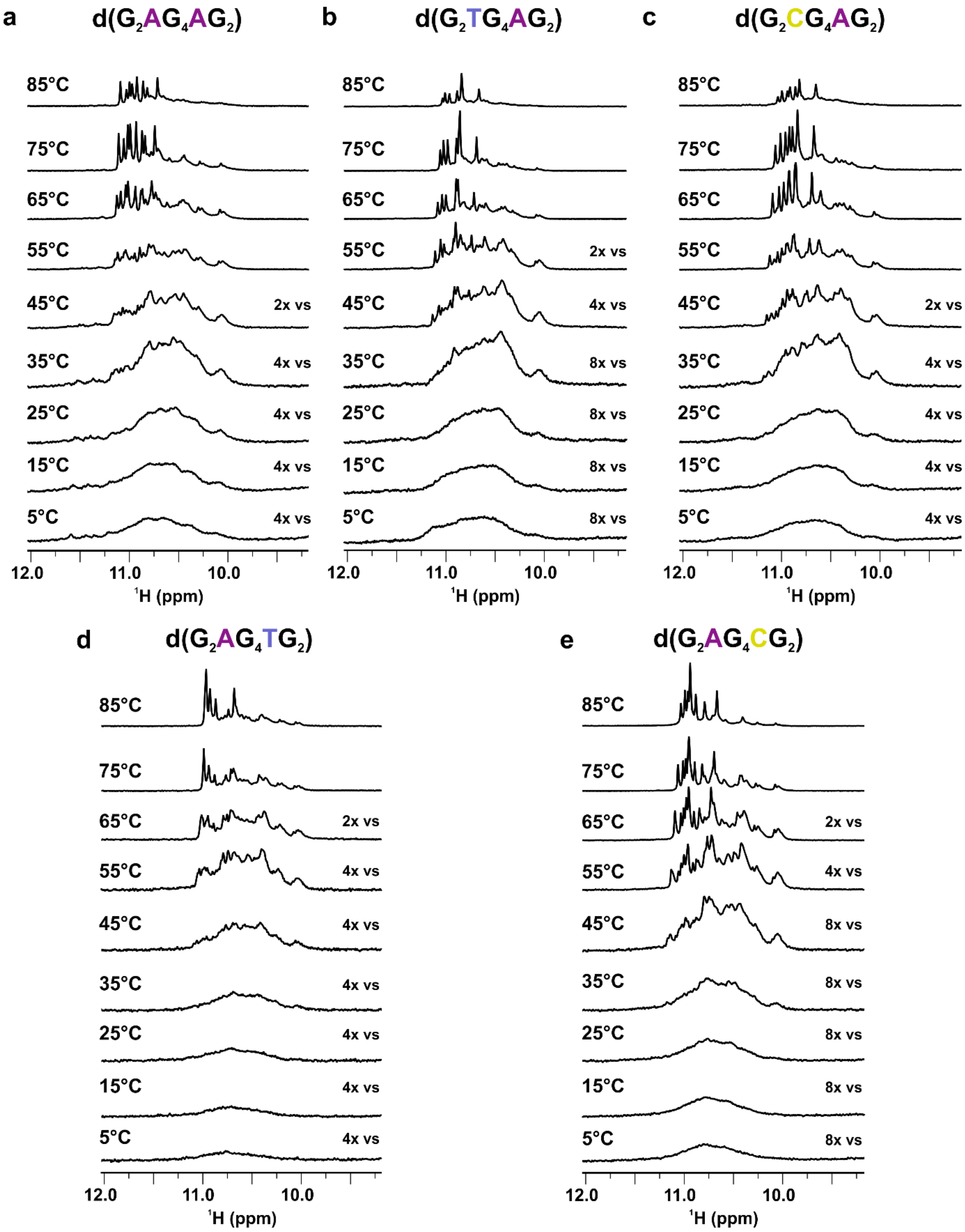

Supplementary Figure 28. NMR melting profiles of G-wires formed by parent and modified oligonucleotides with single loop residue substitutions. H1 regions of 1D ^{1}H NMR spectra of G-wires formed by **(a)** d($G_2AG_4AG_2$), **(b)** d($G_2\underline{T}G_4AG_2$), **(c)** d($G_2\underline{C}G_4AG_2$), **(d)** d($G_2AG_4\underline{T}G_2$) and **(e)** d($G_2AG_4\underline{C}G_2$), acquired between 5 and 85°C as indicated next to the individual spectrum. Spectra were acquired at 600 MHz, 1.0 mM oligonucleotide, 100 mM KCl and 10 mM KPi buffer (pH 6.8) concentrations in 90% H_2O/10% D_2O. Samples were assembled via method 1 (Methods).

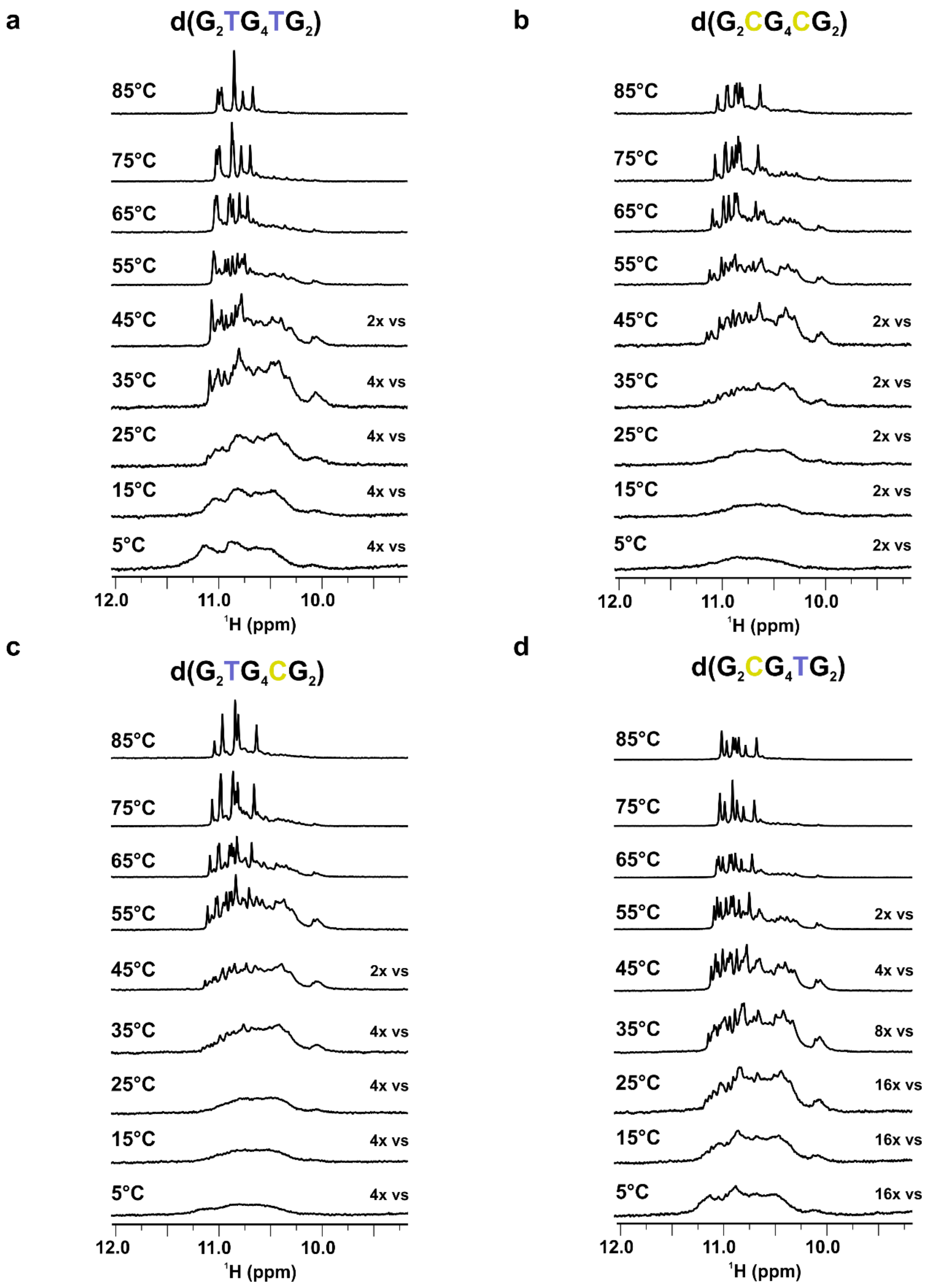

Supplementary Figure 29. NMR melting profiles of G-wires formed by modified oligonucleotides with double loop residue substitutions. H1 regions of 1D ^{1}H NMR spectra of modified G-wires formed by **(a)** d($G_2\underline{T}G_4\underline{T}G_2$), **(b)** d($G_2\underline{C}G_4\underline{C}G_2$), **(c)** d($G_2\underline{T}G_4\underline{C}G_2$) and **(d)** d($G_2\underline{C}G_4\underline{T}G_2$) acquired between 5 and 85°C as indicated next to the individual spectrum. Spectra were acquired at 600 MHz, 1.0 mM oligonucleotide, 100 mM KCl and 10 mM KPi buffer (pH 6.8) concentrations in 90% H_2O/10% D_2O. Samples were assembled via method 1 (Methods).

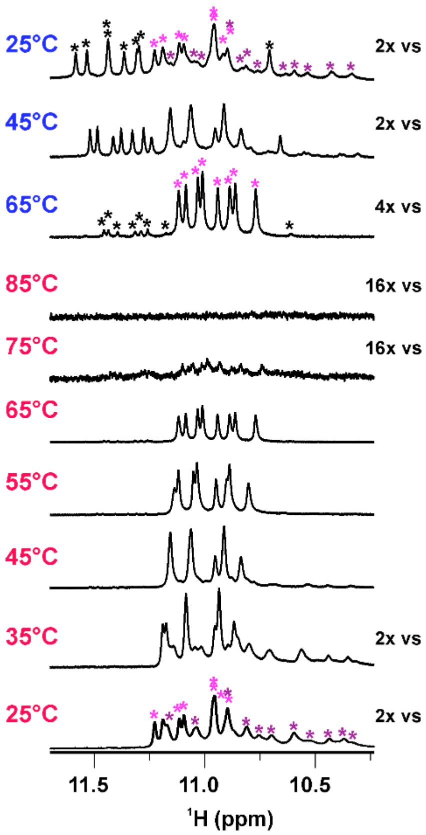

Supplementary Figure 30. NMR melting profile of d($G_2AG_4AG_2$) where only Q2t and Q4t are present in solution. H1 regions of 1D ^{1}H NMR spectra acquired during the heating from 25 to 85°C (temperatures marked with red) and cooling back to 25°C (temperatures marked with blue). During the heating Q4t (labelled with violet asterisks) dissociates into Q2t (labelled with pink asterisks) as indicated by one set of signals observed at 65°C. In the unfolding process, Q2t is the last stable species (i.e., Q1t does not form), which completely unfolds at 85°C in 25 mM KCl. Spectrum, acquired at 65°C during the cooling process reveals two sets of signals corresponding to Q1k and Q2t, which are labelled with black and pink asterisks, respectively. Therefore d($G_2AG_4AG_2$) G-wires have the same folding and refolding mechanism. Spectra were recorded at 600 MHz, 1.0 mM oligonucleotide, 25 mM KCl and 10 mM KPi buffer (pH 6.8) concentrations in 90% H_2O/10% D_2O. Sample was assembled via method 5 (Methods).

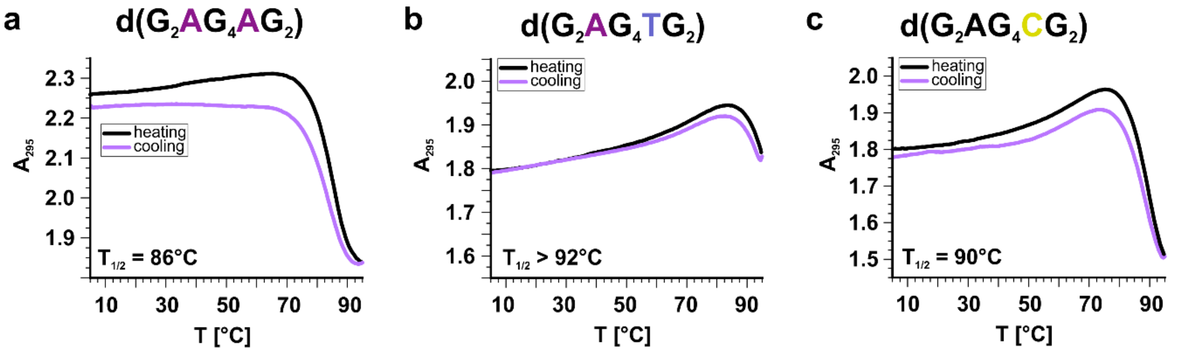

d

Oligonucleotide	$T_{1/2}$ [°C]
d($G_2AG_4AG_2$)	86
d($G_2TG_4AG_2$)	84
d($G_2CG_4AG_2$)	83
d($G_2AG_4TG_2$)	> 92
d($G_2AG_4CG_2$)	90
d($G_2TG_4TG_2$)	92
d($G_2CG_4CG_2$)	89
d($G_2TG_4CG_2$)	88
d($G_2CG_4TG_2$)	90

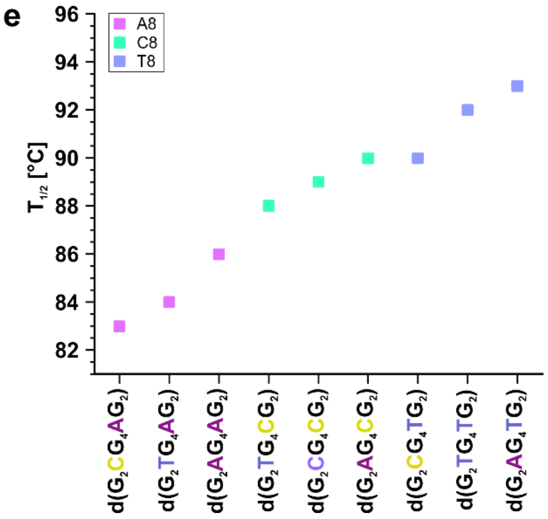

Supplementary Figure 31. The $T_{1/2}$ is mostly influenced by the type of loop residue at position 8. UV melting profiles of G-wires formed by **(a)** parent d($G_2AG_4AG_2$) as well as modified **(b)** d($G_2AG_4\underline{T}G_2$) and **(c)** d($G_2AG_4\underline{C}G_2$) as indicated above individual UV melting profile. Mid-transition temperature ($T_{1/2}$) corresponds to unfolding of Q2t, which is the last stable G-quadruplex in unfolding of G-wires as demonstrated before by NMR melting experiments. Heating and cooling curves are colored with black and violet, respectively. UV melting curves were acquired between 5 and 95°C at 295 nm using the scanning rate of 0.1°C min^{-1}. All samples were assembled via method 1 (Methods). **d** Table shows estimated $T_{1/2}$ derived from heating UV melting curves of different G-wires. **e** Plot shows that loop residue at position 8 has the greatest impact on $T_{1/2}$.

1.11 G-wires with c3 linkers and abasic residues in loops

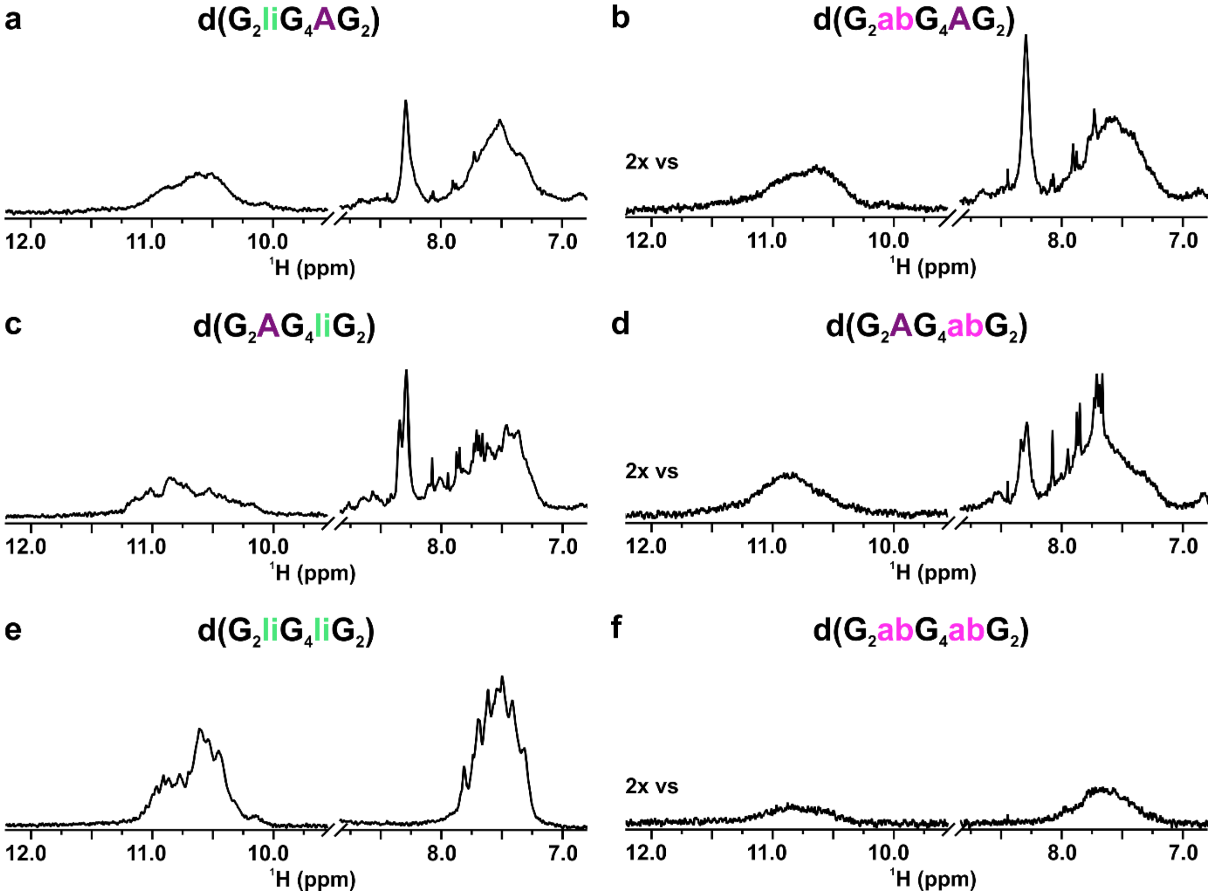

Supplementary Figure 32. Oligonucleotides with c3 linkers and abasic residues in loops form G-wires. H1 and H8 regions of 1D ^{1}H NMR spectra of G-wires formed by **(a)** d(G_2liG$_4$AG$_2$), **(b)** d(G_2abG$_4$AG$_2$), **(c)** d(G_2AG$_4$liG$_2$), **(d)** d(G_2AG$_4$abG$_2$), **(e)** d(G_2liG$_4$liG$_2$) and **(f)** d(G_2abG$_4$abG$_2$). Spectra were acquired at 600 MHz, 25°C, 1.0 mM oligonucleotide and 100 mM KCl concentrations in 90% H_2O/10% D_2O. Samples were assembled via method 1 (Methods).

1.12 Structural ensembles of model Q4t G-quadruplexes

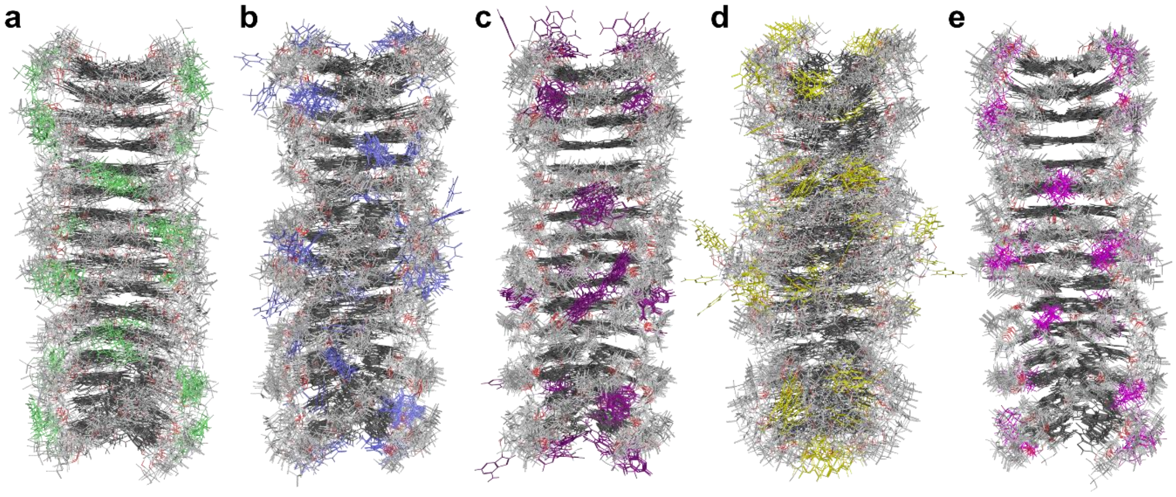

Supplementary Figure 33. Structural ensembles of 10 structures with the lowest energy and the lowest number of restraint violations. A family of 10 Q4t structures formed by **(a)** d($G_2\underline{li}G_4\underline{li}G_2$), **(b)** d($G_2\underline{T}G_4\underline{T}G_2$), **(c)** d($G_2AG_4AG_2$), **(d)** d($G_2\underline{C}G_4\underline{C}G_2$) and **(e)** d($G_2\underline{ab}G_4\underline{ab}G_2$). Guanine bases are colored with green. Residues in loops are presented with different colors for clarity.
